# Combination of Hyaluronan and Lyophilized Progenitor Cell Derivatives: Stabilization of Functional Hydrogel Products for Therapeutic Management of Tendinous Tissue Disorders

**DOI:** 10.3390/pharmaceutics13122196

**Published:** 2021-12-19

**Authors:** Alexis Laurent, Alexandre Porcello, Paula Gonzalez Fernandez, Annick Jeannerat, Cédric Peneveyre, Philippe Abdel-Sayed, Corinne Scaletta, Nathalie Hirt-Burri, Murielle Michetti, Anthony de Buys Roessingh, Wassim Raffoul, Eric Allémann, Olivier Jordan, Lee Ann Applegate

**Affiliations:** 1 Applied Research Department, LAM Biotechnologies SA, CH-1066 Épalinges, Switzerland; annick.jeannerat@lambiotechnologies.com (A.J.); cedric.peneveyre@lambiotechnologies.com (C.P.); 2 Regenerative Therapy Unit, Lausanne University Hospital, University of Lausanne, CH-1066 Lausanne, Switzerland; philippe.abdel-sayed@chuv.ch (P.A.-S.); corinne.scaletta@chuv.ch (C.S.); nathalie.burri@chuv.ch (N.H.-B.); murielle.michetti@chuv.ch (M.M.); lee.laurent-applegate@chuv.ch (L.A.A.); 3 Manufacturing Department, TEC-PHARMA SA, CH-1038 Bercher, Switzerland; 4 School of Pharmaceutical Sciences, University of Geneva, CH-1206 Geneva, Switzerland; alexandre.porcello@unige.ch (A.P.); paula.gonzalezfernandez@unige.ch (P.G.F.); eric.allemann@unige.ch (E.A.); olivier.jordan@unige.ch (O.J.); 5 Institute of Pharmaceutical Sciences of Western Switzerland, University of Geneva, CH-1206 Geneva, Switzerland; 6 DLL Bioengineering, Discovery Learning Program, STI School of Engineering, École Polytechnique Fédérale de Lausanne, CH-1015 Lausanne, Switzerland; 7 Children and Adolescent Surgery Service, Lausanne University Hospital, University of Lausanne, CH-1011 Lausanne, Switzerland; anthony.debuys-roessingh@chuv.ch; 8 Lausanne Burn Center, Lausanne University Hospital, University of Lausanne, CH-1011 Lausanne, Switzerland; wassim.raffoul@chuv.ch; 9 Plastic, Reconstructive, and Hand Surgery Service, Lausanne University Hospital, University of Lausanne, CH-1011 Lausanne, Switzerland; 10 Center for Applied Biotechnology and Molecular Medicine, University of Zurich, CH-8057 Zurich, Switzerland; 11 Oxford OSCAR Suzhou Center, Oxford University, Suzhou 215123, China

**Keywords:** cell therapies, hyaluronic acid, hydrogels, progenitor cells, regenerative medicine, stabilization, tendinopathies, viscoelasticity

## Abstract

Cultured progenitor cells and derivatives have been used in various homologous applications of cutaneous and musculoskeletal regenerative medicine. Active pharmaceutical ingredients (API) in the form of progenitor cell derivatives such as lysates and lyophilizates were shown to retain function in controlled cellular models of wound repair. On the other hand, hyaluronan-based hydrogels are widely used as functional vehicles in therapeutic products for tendon tissue disorders. The aim of this study was the experimental characterization of formulations containing progenitor tenocyte-derived APIs and hyaluronan, for the assessment of ingredient compatibility and stability in view of eventual therapeutic applications in tendinopathies. Lyophilized APIs were determined to contain relatively low quantities of proteins and growth factors, while being physicochemically stable and possessing significant intrinsic antioxidant properties. Physical and rheological quantifications of the combination formulas were performed after hydrogen peroxide challenge, outlining significantly improved evolutive viscoelasticity values in accelerated degradation settings. Thus, potent effects of physicochemical protection or stability enhancement of hyaluronan by the incorporated APIs were observed. Finally, combination formulas were found to be easily injectable into ex vivo tendon tissues, confirming their compatibility with further translational clinical approaches. Overall, this study provides the technical bases for the development of progenitor tenocyte derivative-based injectable therapeutic products or devices, to potentially be applied in tendinous tissue disorders.

## 1. Introduction

Hyaluronan-based hydrogels have historically been widely used in cutaneous and musculoskeletal regenerative medicine, due to an excellent biocompatibility, a high polyvalence in formulation possibilities, a wide tunability, and the potential for designing of diverse functionalities [1,2,3,4,5]. Specifically, such hydrogel therapeutic products or devices have been applied for the management of sub-critical cartilage, bone, and tendon tissue affections such as acute trauma, degenerative pathologies, and related symptoms, or of skin conditions and wounds [6,7,8,9,10,11,12]. Extensive evidence has been reported around the various therapeutic and the ancillary uses of hyaluronic acid (HA) and its derivatives in diverse categories of commercial products comprising pharmaceuticals, complex biologicals, cosmeceuticals, and cosmetics [13,14,15,16,17]. Of note, appropriate HA-containing formulations have been used alone or as delivery vehicles yielding small molecules, proteins, or cells for musculoskeletal disorders and related symptoms such as management of tendinopathies, osteoarthritis, and some adjuvant post-surgical applications [1,18,19,20,21,22]. Multiple simple (e.g., sugars, natural extracts) or complex (e.g., chemical linkers, vitamins, hyaluronidase inhibitors) hydrogel ingredients and manufacturing methods have been investigated for the promotion of the principal or the ancillary intended therapeutic effects and for product stability enhancement [23,24,25,26,27,28,29,30,31,32,33,34]. Furthermore, HA-based hydrogels have been described for the therapeutic delivery of multiple biological components such as stem cells, progenitor cells, autologous platelet-rich plasma (PRP), or specific growth factors [35,36,37,38,39,40].

Various cultured primary progenitor cell types manufactured through optimized cell bank systems have been extensively studied and preclinically or clinically applied in diverse applications of cutaneous and musculoskeletal tissue engineering, notably for the regeneration promotion of skin, cartilage, and tendons under the Swiss progenitor cell transplantation program [41,42,43]. Extensive selection optimization of the therapeutic starting materials had enabled the development of specific pre-natal tissue-derived progenitor cell types for tissue engineering purposes [44]. Such cellular active pharmaceutical ingredients (API) were then characterized and qualified with regard to quality and stability in view of the allogeneic homologous management of traumatic or degenerative tissular affections in human and/or veterinary regenerative medicine over the past twenty years [44,45,46,47,48]. Among these robust cell sources, clinical-grade progenitor tenocyte cell banks (i.e., constituted by the FE002-Ten cell types) have been considered for large-scale API manufacture for eventual tissue engineering purposes [1,42,44,47]. While the historical cytotherapy approaches have relied on the allogeneic uses of cultured and viable cellular APIs, formulated in standardized transplant products (TrSt), recent findings have indicated that specific progenitor cell derivatives (e.g., cell lysates, lyophilizates) retain conserved functions in controlled in vitro cellular models of wound healing [45,46,47,48]. Therefore, in alignment with the widespread current developments of cell-based cell-free therapeutic products which may contain cell lysates, exosomes, or cell secretomes, alternative approaches to cell therapies and standardized transplants have been investigated, for the optimization of product quality, stability, development costs, and regulatory classification processes [36,37,38,49]. An important milestone consisted in obtaining effective cell-derived off-the-shelf API or product prototypes, to be appropriately registered as biologicals or as medical devices (MD) for tissular repair or tissue regeneration promotion [48]. Based on such proceedings, we now propose novel injectable combination formulations incorporating hyaluronan and progenitor tenocyte-derived APIs, based on their respective technical and quality advantages stated hereabove.

The overall objective of this study was the experimental in vitro characterization of the selected formulas incorporating progenitor tenocyte-derived APIs (i.e., lyophilized progenitor tenocytes or lyophilized progenitor tenocyte lysates) in linear HA-based hydrogels, for the assessment of ingredient compatibility and stability in view of the eventual therapeutic applications in tendinopathies. Specific aims comprised the assessment of the putative protective or stabilizing effects of the APIs on evolutive key or critical attributes of the product prototypes, such as the physical and rheological product behaviors in accelerated degradation assays and the corresponding endpoint formulation viscoelastic properties. The present study set forth an approach focusing on the interactions between the lyophilized progenitor cell-derived APIs and the functional HA-based hydrogels, for optimal stabilization of the combination product prototypes. Specifically, the formulation rheological behaviors were assessed after a hydrogen peroxide challenge to mimic the reactive oxygen species (ROS)-mediated hydrogel degradation. Furthermore, specific stability parameters, the protein and growth factor contents, and the intrinsic antioxidant activities of the considered progenitor tenocyte-derived APIs were determined. Significant effects of physicochemical protection or stability enhancement of the hydrogels by the specified APIs were described and discussed from technical and regulatory points of view. Finally, combination product prototypes were qualified in terms of syringeability and injectability in ex vivo tendon tissues, to confirm their compatibility with the further translational clinical approaches. Overall, the present study provides the technical bases for the industrial development of progenitor tenocyte derivative-based injectable hydrogel products or devices, to potentially be applied therapeutically in tendinous tissue disorders or for adjuvant post-surgical use.

## 2. Materials and Methods

### 2.1. FE002-Ten Primary Progenitor Tenocyte Cell Sourcing and In Vitro Cell Culture Initiation

A regulated organ donation at 14 weeks of gestation (i.e., FE002 donation, performed in 2009) served for the procurement of the tissular starting materials used for the establishment of the primary progenitor tenocyte cell source (i.e., FE002-Ten cell type) used in the investigations presented herein. The FE002 organ donation was procured in view of eventual clinical work in tissue engineering and was appropriately included in an ad hoc transplantation program for clinical cell banking (i.e., registered with the Swiss federal health authorities since 2008). Full informed consent was obtained and confirmed for the organ donation and for the inclusion in the ad hoc progenitor cell transplantation program, including for all the potential subsequent research and the development applications. In addition to the extensive donor medical history screening analysis, cytogenetic analyses, and histopathological investigations of the donated tissues, the donor was serologically tested twice (i.e., at the time of the donation and three months later) for specified pathogens (i.e., CMV, EBV, HBsAg, HBV, HCV, HIV-1, HIV-2, HSV, HTLV-1, HTLV-2, S-West Nile virus, *Toxoplasma gondii*, *Treponema pallidum*). Among other primary cell sources, primary progenitor tenocytes were isolated in vitro from the FE002 organ donation following a validated protocol, approved by the local State Ethics Committee (i.e., University Hospital of Lausanne–CHUV, Ethics Committee Protocol #62/07: “Development of fetal cell banks for tissue engineering”, August 2007). The FE002 organ donation was appropriately registered under the Swiss progenitor cell transplantation program and the related progenitor cell biobank, complying with the laws and with the regulations within both programs [44]. The isolated tendon tissue biopsies were mechanically and/or enzymatically processed for the in vitro culture initiation of fibroblastic adherent primary progenitor tenocytes (i.e., FE002-Ten cell types) in good manufacturing practice (GMP)-compliant manufacturing suites, as described previously [44]. Briefly, the procured tendon tissue samples were thoroughly washed in conserved phosphate-buffered saline (PBS) buffer (Bichsel, Interlaken, Switzerland), further dissected, and appropriately processed and conditioned for adherent primary progenitor cell proliferation initiation, as previously detailed [44]. After the initial addition of adequate proportions of the cell culture medium (i.e., Dulbecco’s modified Eagle medium, DMEM, supplemented with 10% *v*/*v* fetal bovine serum, FBS, Gibco™ and Invitrogen™, respectively, ThermoFisher Scientific, Waltham, MA, USA), the planted cell culture vessels were incubated at 37 °C in humidified incubators under 5% *v*/*v* CO_2_. Following the regular iterative cell culture medium exchange procedures, preliminary progenitor cell cultures were harvested by trypsinization (i.e., 0.05% trypsin-EDTA, Gibco™, ThermoFisher Scientific) and were further used to perform in vitro monolayer sub-cultures of FE002-Ten cells following the defined ad hoc technical specifications [44]. Following appropriate maintenance and harvest of the primary cell sub-cultures, the obtained biological materials were cryopreserved in individual polymeric vials in a DMSO-containing cryopreservation solution for the establishment of FE002-Ten parental cell banks (PCB) at passage level 1. After appropriate testing, qualification, and quarantine release of the cryopreserved FE002-Ten PCB cellular material lots, these were used as the starting materials in defined serial expansion workflows, in order to establish FE002-Ten master cell banks (MCB) and the FE002-Ten working cell banks (WCB) used for the present study.

### 2.2. FE002-Ten Primary Progenitor Tenocyte Cell Banking and Bulk API Starting Material Manufacture

In order to generate adequate and sufficient quantities of the cellular materials for the present study, several FE002-Ten WCB vials at passage level 6 were initiated and were used as the manufacturing starting materials in order to produce the bulk API starting material batches. Briefly, the selected cryopreserved FE002-Ten WCB vials were removed from liquid nitrogen storage and were rapidly transferred to the manufacturing suite on dry ice. Following rapid thawing under controlled mechanical agitation, the contents of the vials were gently resuspended in warmed complete cell culture medium (i.e., DMEM, Gibco™, Waltham, MA, USA, with 10% *v*/*v* FBS, Sigma-Aldrich™, St. Louis, MO, USA, and 5.97 mM L-glutamine, Gibco™) in sterile centrifuge tubes (Falcon^®^, Corning^®^, Glendale, AZ, USA). The tubes were then centrifuged at (280 ± 10)× *g* at ambient temperature for 10 min, before the cellular materials were resuspended in the pre-warmed complete cell culture medium. The progenitor cell suspension titers and the relative cellular viability were determined by hemocytometer (NanoEnTek, Seoul, Korea) counts using Trypan blue exclusion dye (Sigma-Aldrich™, Saint Louis, MO, USA), and the cell suspensions were used to homogenously seed the appropriate amounts of sterile non-coated cell culture vessels (Greiner Bio One, Frickenhausen, Germany). Cell seeding was performed at a relative viable density of 1.5 × 10^3^ cells/cm^2^, and the cell culture vessels were incubated at 37 °C in humidified incubators under 5% *v*/*v* CO_2_. Thereafter, the cell culture medium was exchanged twice weekly. The cell cultures were macroscopically and microscopically examined at each medium exchange procedure, for the confirmation of in vitro cell adhesion, cell proliferation, adequate proliferative cellular morphology maintenance (i.e., characteristic spindle-shape cellular morphology), and the absence of observable extraneous agent contamination. Conditioned medium samples were removed and were processed as appropriate for mycoplasma absence verification and were sent for processing to an external laboratory (Eurofins, Ebersberg, Germany). Once the optimal cell monolayer confluency was attained (i.e., >95%), the cell cultures were rinsed with PBS (Bichsel, Switzerland) and were harvested (i.e., TrypLE™ 1 × dissociation reagent, Gibco™). Following collection and pooling of all the harvesting suspensions, the cell suspension titers were determined as previously detailed. The cell population doubling values (PDV) were determined for the FE002-Ten bulk manufacturing batches using the following equation:

PDV = 3.32 × [log_10_ (*N*/*n*)], (1)

where *N* was the total viable cell count determined at the time of confluent cell harvest and *n* was the total viable cell count at the time of seeding of the cells in the culture vessels. The cell population doubling times (PDT) were then determined for the FE002-Ten bulk manufacturing batches using the following equation:

PDT = (*T*/PDV) (2)

where *T* was the total cell culture vessel incubation time expressed in hours from the time of cell seeding to the time of initiation of the cell harvest procedure and PDV was the population doubling value determined previously for the specific considered cellular expansion round. The cell suspensions were then centrifuged at (280 ± 10)× *g* at ambient temperature for 10 min, before the collected cells were resuspended in PBS (Bichsel, Switzerland) for rinsing of the residual cell culture medium components. The cell suspensions were centrifuged again as described hereabove, before the whole cell rinsing procedure was repeated a second time. Eventually, the PBS supernatants were removed from the centrifugation tubes and the resulting FE002-Ten cell pellets were appropriately stored following a defined process in −80 °C ultralow temperature freezers until further processing.

### 2.3. FE002-Ten Bulk Cellular Material Lot Proteomic Characterization by Multiplex Analyses

In order to characterize the considered APIs in terms of the protein and the growth factor quantitative contents, specific multiplex analyses were performed. The proteomic characterization of the bulk FE002-Ten cellular materials was performed, using a specialized service platform (Eve Technologies, Calgary, AB, Canada), on the bulk progenitor cell lysates (i.e., following thermal cell disruption by freeze-thaw cycles). The samples were prepared from the bulk cell pellets by constituting homogenous stock FE002-Ten cell suspensions in PBS (i.e., 10^7^ cells/mL, Bichsel, Switzerland), which were then thermally disrupted and were centrifuged at 13,000× *g* at ambient temperature for 5 min. The supernatants were isolated and were conditioned in low protein-binding Eppendorf tubes (Eppendorf, Hamburg, Germany). The total protein contents in the unfractionated cell lysate samples and in the isolated cell lysate supernatants were determined using a BCA assay kit (ThermoFisher Scientific) following the manufacturer’s protocol and related technical specifications. The FE002-Ten cell lysate supernatant samples were stored at −80 °C and were subsequently sent frozen on dry ice to Canada for the proteomic analyses. The various kits and related workflows (Discovery Assay^®^, Eve Technologies, Calgary, AB, Canada) used for the proteomic analyses of the samples comprised the human angiogenesis array and growth factor 17-plex array, the human cytokine/chemokine 65-plex panel, the human soluble cytokine receptor 14-plex array, the human MMP and TIMP panel for cell cultures and non-blood samples, the multi-species cytokine 3-plex TGF-beta array, and specific assays for IFNα, IFNβ, IFNω, or IL-29.

### 2.4. FE002-Ten Primary Progenitor Tenocyte Derivative Lyophilized API Manufacturing Process

In order to obtain the stable progenitor cell derivative APIs for further studies, two adapted lyophilization workflows were applied. Starting from the FE002-Ten bulk cellular material lots manufactured and characterized as described hereabove, the progenitor tenocyte derivative APIs were manufactured in the form of FE002-Ten lyophilized cell lysates (i.e., referred to hereafter as “cell lysate APIs”) and in the form of FE002-Ten whole cell lyophilizates (i.e., referred to hereafter as “whole cell APIs”). Firstly, the FE002-Ten progenitor tenocyte lysates were obtained by thermal cell disruption applied to the bulk cellular materials, using cyclic transfers from liquid nitrogen to a 37 °C waterbath (i.e., 3 min per incubation step, 3 transfers). For all these freeze-thaw cycles, the bulk cell pellets were first resuspended in sterile PBS (Bichsel, Switzerland) at a final concentration of 10^7^ cell equivalents per mL. The cell lysates were then processed immediately or were stored at –80 °C until further use. Secondly, the bulk progenitor cell pellets or the obtained cell lysates were parallelly reconstituted in a sterile (i.e., filtered on 0.22 µm porous membranes, Stericup^®^, Millipore^®^, Merck, Darmstadt, Germany) lyo-protective solution (i.e., PBS-injectable water mix, Bichsel, Switzerland, with 8.0% *m*/*v* saccharose, PanReac AppliChem, Darmstadt, Germany, and 2.0% *m*/*v* dextran 40,000, Pharmacosmos, Wiesbaden, Germany). The final progenitor cell concentration (i.e., expressed as equivalent cell units for the cell lysates) was of 2 × 10^6^ units/mL in the ad hoc lyo-protective solution. The corresponding lyophilizate placebos were prepared using only the solvents and the polymeric components of the lyo-protective solution. The resulting suspensions and solutions were then aseptically dispensed in 2R lyophilization vials (i.e., 2 mL ISO Schott type I glass vials, Schott, Mainz, Germany), with final filling volumes of 0.75 mL/vial. The vials were stoppered (i.e., 13 mm FluoroTec^®^ laminated Stoppers, Adelphi Healthcare Packaging, Haywards Heath, UK) at half position and placed in AdaptiQ^®^ nests (AdaptiQ^®^ Clip nest, 100 vials/nest, Schott, Germany), which were subsequently placed in ad hoc lyophilization bags (Lyoprotect^®^ single-use bag, 420 mm × 460 mm, with LPMU VS46 bag closure systems, Teclen, Oberpframmern, Germany). The resulting conditioned product packs were then frozen in a defined process at −20 °C until further processing. Subsequently, the conditioned samples were lyophilized in a benchtop lyophilizator (LyoBeta Mini, Telstar, Terrassa, Spain). The sample loading step was performed at −30 °C chamber set temperature. An additional sample cooling step was performed for 2 h at −45 °C chamber set temperature. A vacuum of 0.08 mbar was established and the primary drying step was automatically performed over 39 h using a ramp mode from −45 °C to 25 °C chamber set temperature. A secondary drying step was then performed at maximal vacuum over 9 h using a ramp from 25 °C to 20 °C chamber set temperature. Adequate water removal at the end of the lyophilization cycle was confirmed by the superposition of the two recorded pressure curves, produced from continuous measurements by the Baratron capacitance manometer and the Pirani gauge, respectively. The sample vials were then automatically fully stoppered and finally manually sealed using polymer-aluminum crimps-seals (Adelphi Healthcare Packaging, Haywards Heath). The obtained FE002-Ten lyophilizates (i.e., whole cell API and cell lysate API) were appropriately labelled and stored at 4 °C until further use.

### 2.5. Lyophilized FE002-Ten Primary Progenitor Tenocyte Derivative API Characterization

In order to assess the quality and the stability of the lyophilized forms of the tenocyte-derived APIs, various characterization assays were performed as appropriate for the considered pharmaceutical form. Characterization of the lyophilized FE002-Ten progenitor tenocyte derivative APIs was comparatively performed using an adapted semi-quantitative hybrid score scale, as previously presented [48]. Therein, the descriptive and the organoleptic quality controls (QC) comprised sample photographic recording, the grading of lyophilizate cake presence, batch uniformity, the cake aspect (i.e., cake color, structure, apparent density, finish, topography, collapse, residual material presence, observable particle presence), and the cake behavior (i.e., friability, shrinkage). The qualitative and quantitative quality controls comprised the sample uniformity of mass (i.e., Ph. Eur. Chapter 2.9.5), the particle size distribution analysis (i.e., assessed by laser diffraction, Mastersizer 3000, Malvern Panalytical, Herrenberg, Germany), the relative remaining moisture level (i.e., assessed by the Karl Fisher method, Coulometric KF Titrator Compact C30SD, Mettler Toledo, Greifensee, Switzerland), the cellular structural integrity maintenance (i.e., assessed by contrast phase microscopy for the whole cell samples), and cellular devitalization (i.e., assessed by Trypan blue exclusion dye staining and in vitro recovery assays for the whole cell samples). In order to study the lyophilizate API behavior upon reconstitution, the individual vial contents were rehydrated using a 1% *m*/*v* hyaluronic acid hydrogel (i.e., laboratory grade hyaluronic acid sodium salts, 1.2–1.5 MDa molecular weight (MW), Contipro, Dolní Dobrouč, Czech Republic) in a PBS-injectable water mix (Bichsel, Switzerland). The final reconstituted hydrogel product volume was of 1.5 mL/vial. The composition of the hydrogel was tuned upstream, in order to obtain a final osmolality value of 300 ± 30 mOsm/kg (OsmoTECH^®^ XT, Advanced Instruments, Norwood, MA, USA) in the reconstituted product. The reconstitution time was assessed for the different formulas, as well as the final pH value (SevenCompact Cond meter S230, Mettler Toledo, Switzerland) of the reconstituted hydrogel products. For the long-term study of API stability, the various lyophilizates were parallelly stored at –20 °C, 4 °C, 22 °C, and 37 °C for periods of up to 9 months in the sealed primary packaging vial system.

### 2.6. Lyophilized FE002-Ten Primary Progenitor Tenocyte Derivative API and HA-Based Hydrogel Preparation

In order to confirm the ingredient compatibility and the ease of ingredient combination, the considered APIs were reconstituted in various HA-based hydrogels. Formulation of the combination product prototypes was performed as described for the preliminary lyophilizate API reconstitution assays, by reconstituting the lyophilizate vial contents with the linear HA hydrogels. The final dispensed reconstitution volumes of the hydrogel were of 1.5 mL per vial, bringing the final cell unit concentration to 10^6^/mL and the total cell unit dose to 1.5 × 10^6^/vial. Various linear HA hydrogels were comparatively used for the reconstitution step, comprising 1% *w*/*v* HA with 1.2–1.5 MDa MW, 2% *w*/*v* HA with 1.2–1.5 MDa MW, and 1% *w*/*v* HA with 2.2–2.4 MDa MW (Contipro, Dolní Dobrouč, Czech Republic) in MilliQ water (Millipore^®^, Darmstadt, Germany). For the lyophilizate API reconstitution step, all of the hydrogels were handled with 3 mL Luer-Lok™ syringes (BD, Franklin Lakes, NJ, USA) mounted with 18G blunt-fill needles (BD, USA). Following injection of the HA hydrogels in the lyophilizate API vials and a gentle mechanical dispersion of the rehydrated cake, the combination products were loaded in the Luer-Lok™ syringes using the same 18G blunt-fill needles and were dispensed as appropriate for the various and further characterization experiments.

### 2.7. Combination Product Accelerated Degradation Assays and Rheological Characterization after Hydrogen Peroxide Challenge

The hydrogel accelerated degradation assays were performed to confirm that the combination of the considered ingredients did not result in a negatively impacted product stability. In order to comparatively assess the rheological behaviors of the combination products (i.e., the lyophilized APIs reconstituted with the HA-based hydrogels) under controlled oxidative stress, volumes of 100 µL of hydrogen peroxide (Sigma-Aldrich™, USA) in various initial stock concentrations (i.e., 10–30% *w*/*w*, resulting in final concentrations in the assay items of 2% and 6%, respectively) were added to the product samples (i.e., hydrogel sample volumes of 400 µL). Following an appropriate challenged sample incubation period at 37 °C (i.e., multiple timepoints comprised between 5 min and 24 h), the evolutive and endpoint rheological behaviors of the products were quantitatively determined on a HAAKE Mars Rheometer™ (ThermoFisher Scientific) equipped with a Peltier cone-plate C35 2°/Ti rotor. Appropriate control samples were included at the beginning of the experiments, with non-challenged products (i.e., no addition of H_2_O_2_) and with HA controls. The complex viscosity (η*), storage modulus (G’), and loss modulus (G”) values were assessed and were recorded at 37 °C, respectively, with a constant system oscillatory frequency of 1 Hz. A sample hood was used during all the measurements to minimize the solvent evaporation. The shear stress was set to 3 N/m^2^ in all the experiments, in order to respect the linear viscoelastic region (LVE). The assays were performed in triplicate in the week following the API lyophilization processing and were performed again after 9 months of ingredient and API storage at 4 °C, respectively.

### 2.8. Trolox Equivalent Antioxidant Capacity of Lyophilized APIs

Based on the results of the hydrogen peroxide challenge assays performed on the combination products, further specific investigations were carried out to determine if the considered APIs possessed intrinsic antioxidant properties. To quantify the Trolox equivalent antioxidant capacity (TEAC) of the progenitor tenocyte derivative APIs in the form of lyophilized cell lysates and in the form of whole cell lyophilizates, a Total Antioxidant Capacity Assay Kit (Sigma-Aldrich™) was used with a flat bottom 96-well microtitration plate (Greiner Bio One, Frickenhausen, Germany). The quantitative total antioxidant capacity of the samples, in which Cu^2+^ was reduced by an antioxidant to Cu^+^, was determined colorimetrically. Experimentally, each lyophilizate API sample (i.e., placebo, whole cell, and cell lysate groups) was reconstituted in 300 µL of purified water (Millipore^®^, Burlington, MA, USA). Half of the samples were placed in an ultrasonic bath for 30 min. Following a mechanical mixing step, all the samples were centrifuged a 5400× *g* at ambient temperature for 5 min, and volumes of 20 µL of clear supernatant were transferred to the 96-well plate. Then, equal volumes of 100 µL of the kit reaction mix were added to the samples, and the plate was incubated at ambient temperature for 10 min. The UV absorbance values of the samples were then determined at a wavelength of 570 nm on a microplate reader (Synergy Mx, Biotek, Winooski, VT, USA). The absorbance value at 570 nm was determined to be proportional to the TEAC value in the samples, following the linear Beer–Lambert law. The kit allowed for experimental measurements to be performed within a linear detection range of 1.5–1000 µM in Trolox equivalents, verified by a linear regression curve (i.e., R^2^ = 0.997). The assays were performed in triplicate. The results were presented in absolute values of Trolox equivalents after the correction of the experimental sample dilution factors (i.e., TEAC values were presented as corresponding to an API lyophilizate unit reconstituted with 1.5 mL of the adequate aqueous solvent).

### 2.9. Lyophilized API Physical Characterization by Size Distribution Analysis with Hydrogen Peroxide Challenge

Based on the results of the hydrogen peroxide challenge assays performed on the considered combination products, further specific investigations were carried out to determine the type of effects exerted on the considered APIs by the hydrogen peroxide. Comparative characterization of the particle size distribution in the various diluted API formulations was performed by laser light diffraction using a Mastersizer instrument (Malvern Panalytical, Germany) at ambient temperature. The results were expressed as the span (i.e., polydispersity) and the volume diameters [i.e., D(V,0.1), D(V,0.5), and D(V,0.9)] were calculated directly by the Mastersizer-s V2.19 software (Malvern Panalytical, Malvern, UK). The control samples were prepared by reconstituting the two kinds of lyophilizate APIs using purified water (Millipore^®^). An experimental comparison was performed by reconstituting the various lyophilizate APIs using H_2_O_2_ at a final concentration of 2% *w*/*w* before the characterization of the sample particle size distribution. The delay time between the addition of the H_2_O_2_ to the samples and the measurement on the Mastersizer instrument was of 5 min. The assays were performed with *n* = 6.

### 2.10. Combination Product Syringeability Assessment In Vitro and in Ex Vivo Settings

In order to confirm the that the considered combination products could be administered by injection in clinically compatible administration systems, specific assessments of syringeability and injectability were performed. The force injection profiles of clinically compatible administration systems (i.e., for the treatment of tendinopathies) filled with 250–300 µL of the hydrogel combination products were determined at 22 °C using a set piston speed of 0.5 mm × s^−1^ on a Texture Analyzer TA.XT. Plus instrument (Tracomme, Schlieren, Switzerland). The syringes (Schott TOPPAC^®^ 1 mL long syringe in 100 pieces/nest packs, 5 mm internal diameter, with Luer-Lok™, Schott, Germany) were fitted with 13 mm 27G needles (Needle Concept, Biarritz, France) and were used as the administration system in the experimental setup. The placebos and the API lyophilizates were reconstituted using 1.5 mL of a stock HA hydrogel (i.e., 1.2–1.5 MDa MW, 2% *m*/*v*, Contripro, Czech Republic, in purified water, Millipore^®^) 30 min before the loading of the preparations in the syringes and the further assessment of syringeability and injectability. The final forms of the injectable products (i.e., loaded in the administration systems) were secured on the Texture Analyzer instrument with laboratory tape to exclude any movements other than the required piston downward vertical movement. A first set of measurements was acquired by placing a hydrogel recuperation container under the free needle (i.e., with no counter-pressure). A second set of measurements was acquired with the needle inserted in ex vivo equine tendon tissue samples (i.e., equine superficial digital flexor tendons derived from the food industry, approx. 15 cm in length, 2 cm in width, and 1 cm in depth, Profil Export, Chavrieu Chavagneux, France). The tissues had been harvested, cleaned, and trimmed to standard dimensions, conditioned in plastic bags, and frozen at −80 °C for storage until further use. For the ex vivo experiments, the tendons were thawed overnight at room temperature and were equilibrated at 37 °C for 2 h. The ex vivo tendon tissues were then positioned and secured under the Texture Analyzer instrument so that the needles could be vertically half-way inserted in the tissue, adopting a 45–60° angle with the face of the tendon, and using the first third of the whole tendon length (i.e., to maintain proximity with the ligament junction). For the force injection profile determination step, the evolutive force (N) required to extrude the hydrogels from the administration system was measured and was recorded along the full path of the syringe piston.

### 2.11. Statistical Analyses

For the statistical comparison of average values from two datasets, an unpaired Student’s *t*-test was applied, after the appropriate evaluation of the normal distribution of the data. A *p* value < 0.05 was retained as a base for the statistical significance determination. For the statistical comparison of values from multiple quantitative datasets from experiments where multiple variables applied, a one-way ANOVA test or a two-way repeated measures ANOVA test (i.e., with the Geisser–Greenhouse correction) was performed, and was followed (i.e., when appropriate) by a post-hoc Tukey’s multiple comparison test or was substituted by a Kruskal–Wallis one-way analysis of variance (i.e., for analysis of non-parametric variables/data such as gradings in quality control experiments), which was followed (i.e., when appropriate) by a post hoc Dunn test. A *p* value < 0.05 was retained as a general base for the statistical significance determination. The detailed levels of statistical significance were specified further in the Results Section. The statistical calculations and/or data presentation were performed using Microsoft Excel, Microsoft PowerPoint (Microsoft Corporation, Redmond, WA, USA), and GraphPad Prism v. 8.0.2 (GraphPad Software, San Diego, CA, USA), respectively.

## 3. Results

### 3.1. FE002-Ten Primary Progenitor Tenocyte Bulk Manufacture, Cellular Derivative API Preparation, and API Characterization

**Figure A1:**
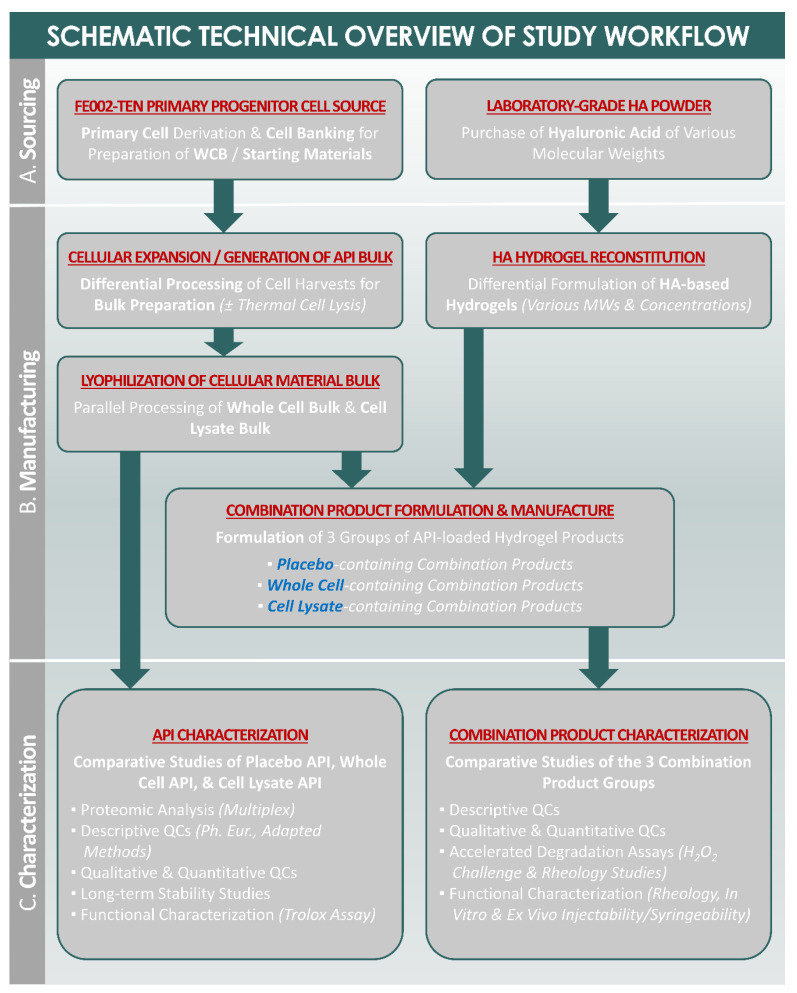
Schematic technical overview of the main steps of the present study. (**A**) The starting materials for the bulk API preparation were processed and the starting materials for the HA hydrogel preparation were purchased. (**B**) Starting materials (i.e., FE002-Ten primary progenitor cells) for the API manufacture were appropriately expanded in vitro, differentially processed in bulk (i.e., with or without thermal cell lysis), and stabilized by lyophilization. Once the HA-based hydrogel vehicles were reconstituted and the progenitor cell derivative APIs were available, the various combination product formulations were prepared. (**C**) Characterization assays and experiments (i.e., descriptive and functional) were carried out on the various forms of the manufactured APIs, as well as on the various combination product formulations. API, active pharmaceutical ingredients; HA, hyaluronic acid; MW, molecular weight; Ph. Eur., European pharmacopoeia; QC, quality control; WCB, working cell bank.

For an enhanced and a facilitated presentation of the experimental study methodology, a schematic and comprehensive technical overview of the different technical steps of the present study is provided as a workflow in the Appendix A (Figure A1). Following the appropriate multi-tiered primary progenitor cell banking in view of the starting material generation, the bulk cellular material batches were derived from the selected FE002-Ten WCB vials at passage level 6. In view of the eventual API stabilization by lyophilization, the cultured progenitor tenocytes were harvested after 15 days of culture incubation, upon attaining 100% confluency (Figures S1–S3). The macroscopic and the microscopic examination of the culture vessels at each medium exchange procedure by two experienced operators allowed for the confirmation of the adequate initial in vitro cell adhesion, the subsequent cell proliferation, the adequate proliferative cellular morphology maintenance (i.e., characteristic spindle-shape cellular morphology), and the absence of observable extraneous agent contamination (i.e., no presence of extraneous particles in the culture medium, no turbidity or drastic pH modifications of the culture medium, Figure S3). The results from the mycoplasma quality control assays demonstrated the absence of the specified (i.e., *M. arginini*, *M. fermentans*, *M. orale*, *M. hyorhinis*, *M. hominis*, *M. genitalium*, *M. salivarium*, *M. synoviae*, *M. pirum*, *M. gallisepticum*, *M. pneumonia*, *M. yeatsii*, *Spiroplasma citri*, *Acholeplasma laidlawii*) and of non-specified mycoplasma species. The mean primary progenitor tenocyte cellular viability was of 98% ± 1% after the cell harvest procedure. The mean PDV was of 4.32 ± 0.54 and the mean PDT was of 84.2 h ± 4.9 h for the considered FE002-Ten bulk cellular material production batches. The total protein quantification by BCA assay, performed on pooled bulk cellular materials, indicated mean values of 1891 µg/mL ± 115 µg/mL (i.e., in the complete unfractionated FE002-Ten cell lysates) and mean values of 1094 µg/mL ± 28 µg/mL (i.e., in the harvested FE002-Ten cell lysate supernatants) of total proteins in the samples, respectively. The multiplex proteomic analyses of the bulk FE002-Ten cell lysate supernatants allowed for the identification of around 100 proteins within the limits of the experimental setup (Table 1, Tables S4 and S5).

The 20 most abundant detected proteins in the bulk cellular material samples consisted in enzymes, soluble receptors, and soluble factors (Table 1). A specific analysis of gene ontology (GO) entries with regard to the molecular functions and the biological processes for the 20 most abundant detected proteins revealed many pathways and processes of interest for tendon tissue homeostasis and repair (Table S4). The harvested and processed bulk cellular materials further served for the manufacture and the characterization of several batches of the API lyophilizates (i.e., using whole cell bulk or unfractionated cell lysate bulk) of 50 vials/batch, along with the corresponding placebo formulations (Figures S4–S7). An ad hoc hybrid results and API grading table was adapted for the quantitative and the semi-quantitative characterization of the lyophilized progenitor tenocyte derivative APIs and of the corresponding placebo formulations (Table 2).

All the investigated lyophilized API parameters were found to be conform to the predefined targets and the specifications or were graded as satisfactory and as optimal (Table 2). The cake color (i.e., cell lysate group) and cake finish (i.e., both sample groups) of the formulations containing progenitor cell derivatives were found to be slightly dull and matte, as compared to the placebo samples, but were assessed as satisfactory (Table 2). The cake structure was graded as satisfactory in both groups, as the obtained API lyophilizates were not perfect cylinders but presented a consistent top concavity, which was reproducible between the groups and between the production batches (Table 2). The cake reconstitution process was simple and rapid, and the final product physical parameters (e.g., pH and osmolality values) were found to conform to the predefined specification ranges (Table 2). It may be noted that the cake color, structure, and finish were assessed as optimal for the placebo sample group, whereas these parameters were mainly graded as satisfactory in the whole cell and in the cell lysate sample groups, respectively (Table 2). This aspect was attributed to the potentially different crystalline structures of the solid phases during freezing and lyophilization of the placebo group as compared to the respective API groups.

The quantitative results of lyophilizate particle size distribution analysis revealed the presence of a homogenous monomodal particulate population for the whole cell API lyophilizate group (i.e., mean span value of 0.809) and a relatively wider population size distribution for the cell lysate API lyophilizate group (i.e., mean span value of 3.816, Table S1). The results of the long-term stability studies of the lyophilized APIs (i.e., results shown for the whole cell API lyophilizate group) did not evidence significant modifications in the monitored parameters in the defined assay conditions, allowing to conclude to the physicochemical stability of the considered API lyophilizates for a period of up to 9 months (Table 3). The cake reconstitution times were determined to be relatively longer after storage than after lyophilization, yet all the experimental values remained within the defined specification limits (Table 2 and Table 3). It may be noted that for the API stability studies, the used API lots were assessed as homogenous after production and were randomized across all the storage conditions (Table 2). The observed and reported minimal cake modifications in some cases of the endpoint analysis after storage were mostly related to the general cake structure and to the cake friability after the analyses (Table 3). No correlation was outlined between the storage temperature or the storage time and the relatively lower cake resistance described hereabove, and these results of minimal observable cake modification were attributed to the intra-group variability of the analyzed materials.

### 3.2. Lyophilized API-HA Combination Product Characterization in Accelerated Degradation Assays after Hydrogen Peroxide Challenge

**Figure 1:**
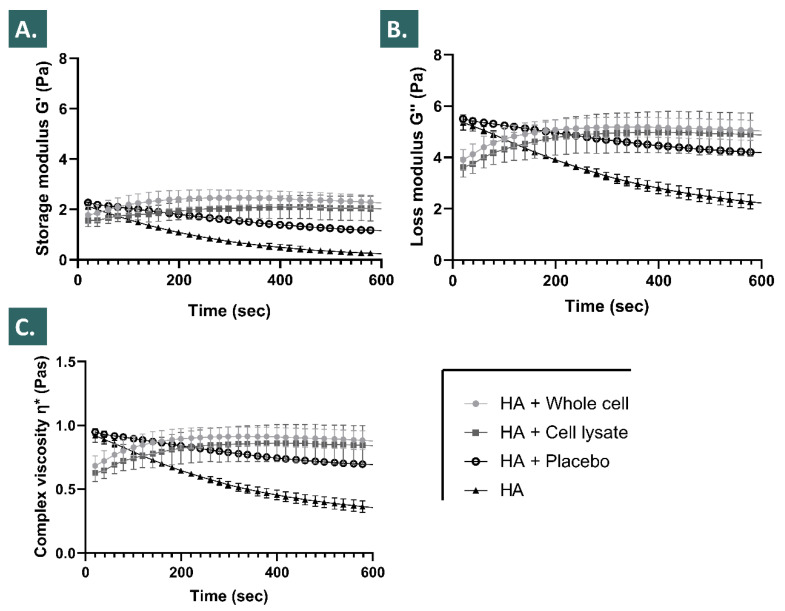
Quantitative and comparative results of the differential and evolutive rheological behaviors, expressed as storage (G’) and loss (G’’) moduli or as complex viscosity (η*) values of the various combination products challenged with 30% *w*/*w* hydrogen peroxide. The assay setup corresponded to a hydrogel formula adapted for an application in tendinopathies, with an oxidative challenge using an extreme quantity of H_2_O_2_. The lyophilizates (i.e., placebos and APIs) were reconstituted in HA one hour before the challenge. The measurements were performed at 37 °C in oscillatory rheology at a frequency of 1 Hz, using a shear stress of 3 N/m^2^ and a total measuring period of 10 min. The delay time between the addition of 100 µL of 30% *w*/*w* H_2_O_2_ to 400 µL of hydrogel samples (i.e., 1.2–1.5 MDa MW HA-based hydrogel at 2.2% *m*/*v*) and the rheological measurements was of 150 s. The results were presented as mean recorded values from the triplicate experiments, assorted to the corresponding standard deviations as the error bars. (**A**) Evolutive storage moduli (G’) values of the various sample groups following the H_2_O_2_ challenge. (**B**) Evolutive loss moduli (G’’) values of the various sample groups following the H_2_O_2_ challenge. (**C**) Evolutive complex viscosity (η*) values of the various sample groups following the H_2_O_2_ challenge. The results outlined significant protective effects of the placebo formulations and of the APIs on the evolutive sample rheological properties. The quantitative results and the statistical analysis results of the endpoint rheological property value comparative assessments are presented in Table S2. HA, hyaluronic acid; MDa, mega Daltons; MW, molecular weight; Pa, Pascals; Pa·s, Pascal seconds; sec, seconds.

**Figure 2:**
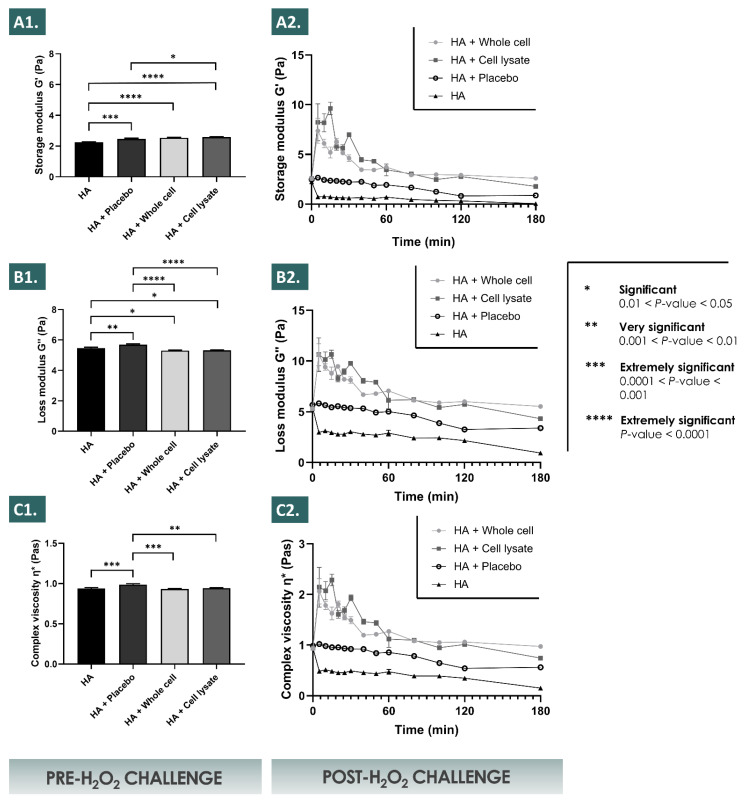
Quantitative and comparative results of the differential initial and of the evolutive rheological behaviors, expressed as the storage (G’) and loss (G’’) moduli or as the complex viscosity (η*), of the combination products challenged with 10% *w*/*w* of hydrogen peroxide. The assay setup corresponded to a diluted hydrogel formula, with an oxidative challenge using a high quantity of H_2_O_2_. The lyophilizates (i.e., placebos and APIs) were reconstituted in 1% *m*/*v* of HA one hour before the challenge. The measurements were performed at 37 °C in oscillatory rheology at a frequency of 1 Hz, using a shear stress of 3 N/m^2^ and a measuring period of 180 min. The delay time between the addition of 100 µL of 10% *w*/*w* H_2_O_2_ to 400 µL of the hydrogel sample and the measurement corresponded to each timepoint, with the challenged samples being incubated at 37 °C before the measurement. The results were presented as the mean recorded values from the triplicate experiments, assorted to the corresponding standard deviations as the error bars. (**A1**) Comparative initial storage moduli (G’) of 1.2–1.5 MDa HA-based hydrogels (1.0% *m*/*v*) containing the placebo or the cell derivative API formulations, with the corresponding HA controls, prior to the H_2_O_2_ challenge. (**A2**) Comparative and evolutive storage moduli (G’) of challenged 1.2–1.5 MDa HA-based hydrogels (1.0% *m*/*v*) containing the placebo or the cell derivative API formulations, with the corresponding HA controls. (**B1**) Comparative initial loss moduli (G’’) of 1.2–1.5 MDa HA-based hydrogels (1.0% *m*/*v*) containing the placebo or the cell derivative API formulations, with the corresponding HA controls, prior to the H_2_O_2_ challenge. (**B2**) Comparative and evolutive loss moduli (G’’) of challenged 1.2–1.5 MDa HA-based hydrogels (1.0% *m*/*v*) containing the placebo or the cell derivative API formulations, with the corresponding HA controls. (**C1**) Comparative initial complex viscosity (η*) values of 1.2–1.5 MDa HA-based hydrogels (1.0% *m*/*v*) containing the placebo or the cell derivative API formulations, with the corresponding HA controls, prior to the H_2_O_2_ challenge. (**C2**) Comparative and evolutive complex viscosity (η*) values of challenged 1.2–1.5 MDa HA-based hydrogels (1.0% *m*/*v*) containing the placebo or the cell derivative API formulations, with the corresponding HA controls. The results outlined significant protective effects of the placebo formulations and of the APIs on the evolutive sample rheological properties. The quantitative results and statistical analysis results of the endpoint rheological property value comparative assessments are presented in Table S3. API, active pharmaceutical ingredients; HA, hyaluronic acid; MDa, mega Daltons; min, minutes; Pa, Pascals; Pa·s, Pascal seconds.

**Figure 3:**
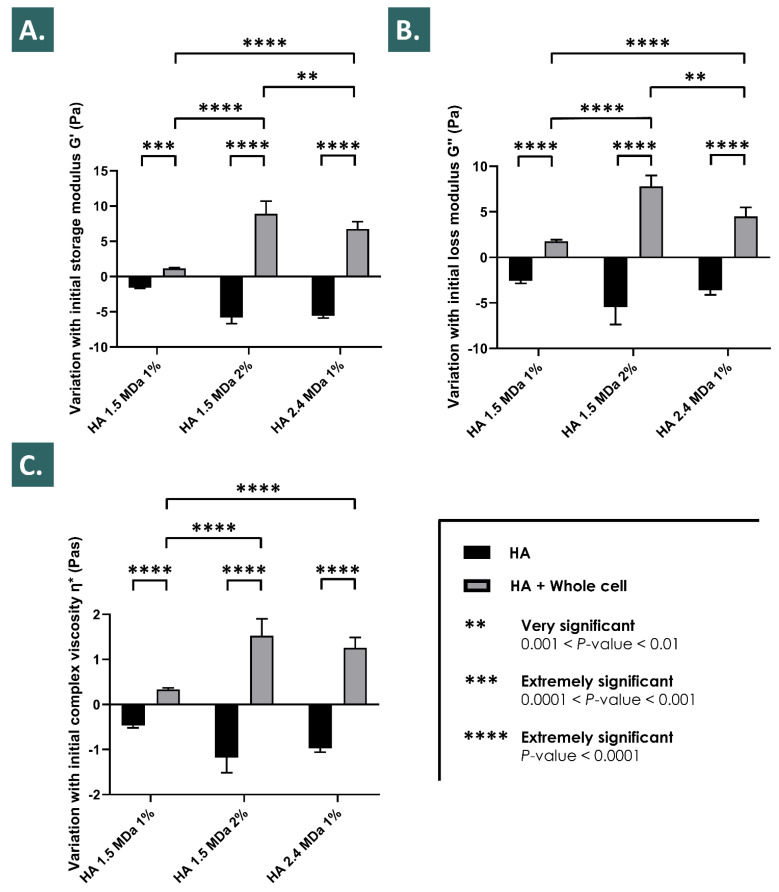
Comparative relative endpoint results displaying the respective differences in the mean storage (G’) and loss (G’’) moduli values or in the mean complex viscosity (η*) values of the various HA-based hydrogels (i.e., different HA concentrations and molecular weights) loaded with the cellular derivatives (i.e., whole cell APIs) and challenged with 10% *w*/*w* H_2_O_2_ for 60 min before the rheological measurements, as compared with the corresponding unloaded and challenged HA control formulations. The results were presented as mean recorded values from the experiments where *n* = 6, assorted to the corresponding standard deviations as the error bars. (**A**) Endpoint relative and comparative assessment of the storage moduli (G’) values of the various loaded and non-loaded challenged formulations, as compared to the corresponding initial storage moduli values. (**B**) Endpoint relative and comparative assessment of the loss moduli (G’’) values of the various loaded and non-loaded challenged formulations, as compared to the corresponding initial loss moduli values. (**C**) Endpoint relative and comparative assessment of the complex viscosity (η*) values of the various loaded and non-loaded challenged formulations, as compared to the corresponding initial complex viscosity values. The results outlined relatively more important endpoint rheological property modifications by the considered APIs for the concentrated HA hydrogels or for the high molecular weight HA hydrogels following the H_2_O_2_ challenge. API, active pharmaceutical ingredient; HA, hyaluronic acid; MDa, mega Daltons; Pa, pascals; Pa·s, Pascal seconds.

The results of the present study showed that in the defined in vitro conditions of the accelerated hydrogel degradation assays using hydrogen peroxide, the inclusion of appropriately formulated progenitor tenocyte derivative APIs resulted in enhanced viscosity values and in enhanced storage moduli across multiple timepoints and multiple assay conditions (Figure 1, Figure 2 and Figure 3).

It was shown that the placebo formulations (i.e., containing saccharose and dextran) procured a significant evolutive viscosity modification effect after the H_2_O_2_ challenge, but that the lyophilized APIs significantly further enhanced said modification across the assessed parameters (Figure 1). Of note, the placebo formulations were characterized by an initial viscosity comparable to that of the HA control group at the initial assay timepoint following the challenge, whereas the subsequent degradation rates (i.e., determined by the absolute values of the slopes of the viscosity curves) of the placebo group were relatively less important when compared to the HA control group (Figure 1). Interestingly, the formulations containing the lyophilized APIs were characterized by relatively inferior initial viscosity values at the initial assay timepoint following the challenge, when compared to the placebo group and to the HA control group (Figure 1). However, the values of the slopes of the corresponding viscosity curves were initially positive, and eventually tended toward zero at the final assay timepoint, suggesting a cancelling of the degrading effects of the H_2_O_2_ on the HA polymeric network by the APIs (Figure 1).

It was experimentally shown that the relatively increased endpoint values of the assessed viscosity parameters (i.e., G’, G’’, and the complex viscosity η*) of the various hydrogels (i.e., various HA molecular weights and concentrations) followed the same trends in the various accelerated degradation assay conditions (i.e., H_2_O_2_ challenge, 2% vs. 6%) after the inclusion of the lyophilized APIs (Figure 1 and Figure 2). Specifically, it was shown that the endpoint rheological property values of the various hydrogels loaded with the APIs were consistently relatively higher as compared to the placebo group and to the HA control group, respectively (Figure 1, Figure 2 and Figure 3). Moreover, it was shown that the relatively increased endpoint viscosity values or the resistance to deformation effects procured by the cellular derivative APIs were on average consistently and relatively more important for the whole cell derivatives than for the lyophilized cell lysates (Figure 1 and Figure 2). This comparative endpoint observation was determined to be a non-significant trend when using 30% *w*/*w* of H_2_O_2_ as the challenge item but was significant when using 10% *w*/*w* of H_2_O_2_ as the challenge item (Figure 1 and Figure 2, Tables S2 and S3). Overall, an additive or a synergistic effect of relative and evolutive rheological property modification was outlined in the various experimental conditions for the different test-items (i.e., placebo components and APIs), which was furthermore confirmed by the use of various hydrogel formulations (Figure 1, Figure 2 and Figure 3). Specifically, the results outlined more important and significant endpoint increases of the rheological property values for the relatively more concentrated hydrogels or for systems with relatively higher molecular weight HA polymers (Figure 3).

Notably, relatively important increases in the sample viscosity values (i.e., G’, G’’, and η*) were transiently observed for the formulations incorporating the APIs (i.e., whole cell and cell lysate lyophilizates) in the first 30 min of the 10% *w*/*w* H_2_O_2_ challenge assays (Figure 2). These effects were determined to be dependent on the presence of the cell derived APIs and of the H_2_O_2_ challenge item, as the transient effects were not observed for the HA control group or for the placebo sample group, respectively (Figure 2).

### 3.3. Characterization of Lyophilized API Intrinsic Total Antioxidant Properties

**Figure 4:**
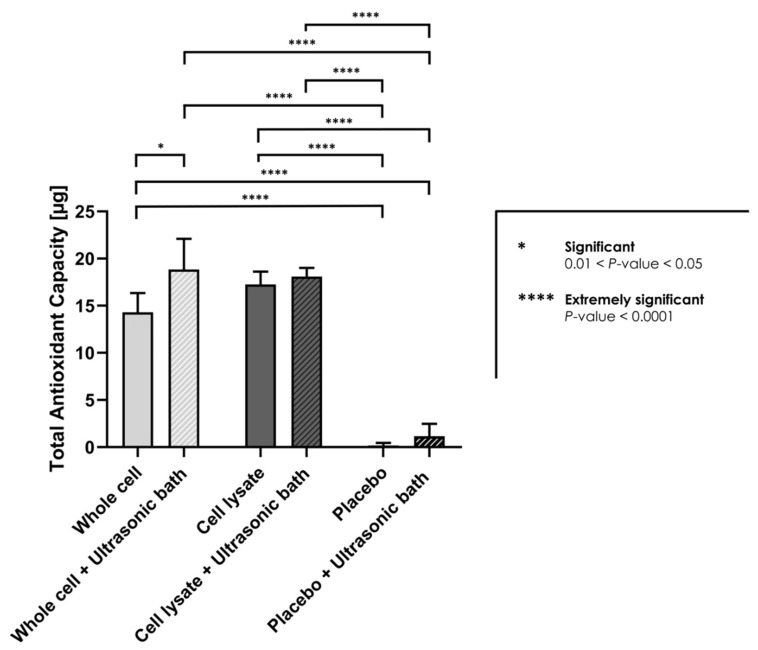
Comparative quantitative absolute values of the Trolox equivalent antioxidant capacity of the cell derivative lyophilizate APIs and of the corresponding placebos submitted to the various sample processing methods. Significant intrinsic antioxidant capacities were recorded for both of the API sample groups. The results did not outline statistically significant differences between the whole cell API and the cell lysate API sample groups. The results did not reveal the presence of measurable TEAC values for the placebo sample groups, suggesting that the antioxidant activity observed in the API groups was intrinsic to said APIs. The TEAC values were expressed in µg and corresponded to the Trolox equivalents for the lyophilized APIs (i.e., one API unit, corresponding to 1.5 × 10^6^ cell unit equivalents/vial) reconstituted appropriately with 1.5 mL of aqueous solvent. The results were presented as the mean recorded values from the triplicate experiments, assorted to the corresponding standard deviations as the error bars. API, active pharmaceutical ingredient; TEAC, Trolox equivalent antioxidant capacity.

The presented results of the HA-based hydrogel sample rheological behaviour modulation by the selected APIs in the various H_2_O_2_ challenge assays allowed to conclude to a significant viscosity modification effect of the considered APIs but were not sufficient to directly conclude to an intrinsic antioxidant effect of the APIs. Therefore, the Trolox equivalent antioxidant capacity of the lyophilized APIs was determined, where significant intrinsic antioxidant properties were experimentally recorded for both of the API sample groups (i.e., mean TEAC values of 14–18 µg Trolox, Figure 4). The results did not outline statistically significant differences between the whole cell and the cell lysate API sample groups, nor between the sample processing methods for the cell lysate API group (Figure 4). However, a significant difference was observed within the whole cell API group when comparing the sample preparation methods. The whole cell API samples submitted to ultrasonication before analysis presented the highest mean TEAC values (i.e., 18.85 ± 3.25 µg Trolox), significantly relatively higher in value as compared to the values of the same non-ultrasonicated whole cell API samples (*p* = 0.0203). No significant TEAC values were detected for the placebo groups, indicating that the determined TEAC values were dependent on the presence of the lyophilized APIs and were intrinsic to said APIs (Figure 4). This was confirmed by the statistical analysis of the differences in mean TEAC values recorded between all the considered API groups and both of the placebo sample groups, where all of the obtained *p* values indicated extremely significant differences (i.e., *p* < 0.0001 in all cases, Figure 4).

### 3.4. Physical Characterization of Particle Size Distribution of Reconstituted APIs Challenged with Hydrogen Peroxide

**Figure 5:**
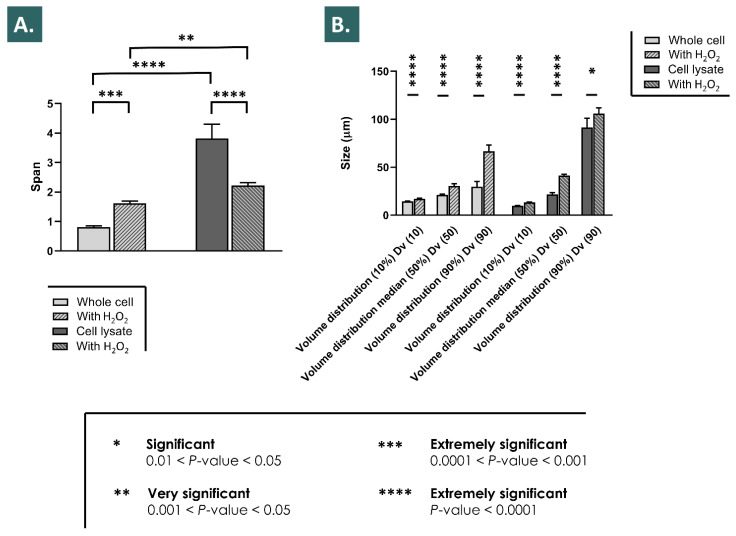
Comparative particle size distribution analysis of reconstituted cellular derivative lyophilizate APIs, with and without hydrogen peroxide challenge. (**A**) The span values revealed a relatively wider particle size distribution for the cell lysate API formulation before the 2% *w*/*w* hydrogen peroxide challenge. The results outlined contrary effects of the challenge item on the whole cell API sample group and on the cell lysate API sample group particle size distributions, respectively. (**B**) The volume distribution parameters showed significant increases in the size of the reconstituted API particles following the hydrogen peroxide challenge. The results were presented as mean recorded values from the experiments where *n* = 6, assorted to the corresponding standard deviations as the error bars. When comparing results expressed as volume distributions between both types of APIs (i.e., corresponding parameters between the API groups, for the challenged or non-challenged samples, respectively), statistically extremely significant (*p* < 0.0001) differences were systematically observed, except for the difference in median volume distribution values Dv (50) of the non-challenged API samples (i.e., whole cells vs. cell lysates), which was found to be not statistically significant. Quantitative results of reconstituted API particle size distribution analysis before hydrogen peroxide challenge are presented in Table S1. API, active pharmaceutical ingredients.

In order to better understand the relatively important initial and transient rise in the recorded rheological property values in the 10% *w*/*w* hydrogen peroxide assay, the isolated effects of H_2_O_2_ on the APIs particle size distribution were studied (Figure 2 and Figure 5). The comparative analysis of the span values before the H_2_O_2_ challenge indicated a relatively wider particle size distribution for the cell lysate API group as compared to the whole cell API group (Figure 5A). However, the effects of the H_2_O_2_ challenge on the span values were specific to each group, with an extremely significant increase in the particle size distribution values for the whole cell API group, and conversely, an extremely significant decrease in the particle size distribution values for the lysate API group (Figure 5A). Regarding the volume distribution values, the relative differences between Dv (10), Dv (50), and Dv (90) values were recorded as being greater in the cell lysate API group before the hydrogen peroxide challenge, in accordance with the corresponding span value (Figure 5B). Regarding the impacts of H_2_O_2_ on the volume distribution values in both groups, similar results were recorded, with significant relative increases in the volume distribution medians (i.e., Dv (50) values) in both groups (Figure 5B). Overall, significant effects of particle size distribution modification were observed by laser light diffraction following the challenge of the reconstituted APIs with 2% *w*/*w* H_2_O_2_, providing additional information about the physical behavior of the biological derivatives in the accelerated combination product degradation assays presented hereabove (Figure 2 and Figure 5). Additionally, it is to note that after addition of the H_2_O_2_ and before the dilution of the samples for laser light diffraction analysis, an increase in the observed presence of bubbles was noted in both hydrogel samples groups (results not shown).

### 3.5. Characterization of Combination Product Syringeability In Vitro and Ex Vivo

**Figure 6:**
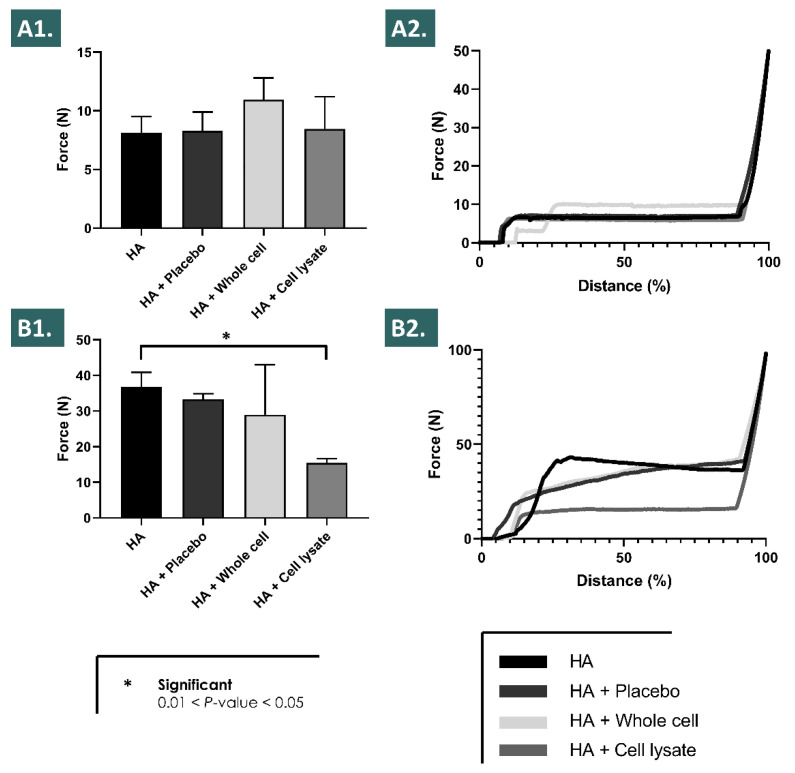
Results of in vitro and ex vivo syringeability assays of the combination products, performed in triplicate measurements at ambient temperature. (**A1**) Comparative mean injection forces required to extrude the product formulas through the specified administration system. The individual values were obtained over a distance of 1 mm in the linear phase of the force injection profile, and the mean recorded forces were assorted with the corresponding standard deviations as the error bars. (**A2**) Comparative force injection profiles of the product formulas with injection into air. (**B1**) Comparative mean injection forces required to extrude the product formulas through the specified administration system into an ex vivo equine tendon tissue sample. The individual recorded values were obtained over a period of 1 s at half distance on the force injection profile, and the mean recorded forces were assorted with the corresponding standard deviations as the error bars. (**B2**) Comparative force injection profiles of the product formulas with injection into an ex vivo equine tendon tissue sample. HA, hyaluronic acid; N, Newtons.

In order to determine if the considered lyophilized API-HA hydrogel combination products could technically be used in standard therapeutic product delivery systems, in vitro and ex vivo syringeability and injectability assays were performed following the appropriate API and product reconstitution steps (Figures S8–S10). The in vitro experimental results outlined that all the considered product formulations could be relatively easily extruded through a 27G needle using mean forces < 10 N, without any statistically significant difference detected between the test items (Figure 6A). Along with a horizontal plateau in the corresponding force injection profile, this aspect contributed to confirm that the included APIs did not cause tangible modifications in the formula rheological behavior (i.e., in the absence of strong oxidants) which could have compromised the syringeability, as the addition of whole cells did not induce an increase in the required mean extrusion force (Figure 6A). Furthermore, repetition of the syringeability assays in the ex vivo setting with injection of the product in equine tendon tissue samples highlighted the technical possibility of relatively easily injecting the considered combination products in situ (Figure 6B). Higher intergroup assay variability was recorded in the ex vivo setting, along with an increased required mean injection force (Figure 6B).

However, said injection force did not exceed 50 N in any of the ex vivo experimental conditions, denoting an acceptable syringeability or injectability of all the considered formulas in the clinically relevant and specified administration systems (Figure 6B).

## 4. Discussion

### 4.1. Lyophilized Progenitor Tenocyte Derivative APIs Present Extensive Physicochemical Stability and Contain Low Quantities of Multiple Proteins

The various accelerated and long-term stability studies performed on the lyophilized APIs have demonstrated an extended stability thereof for at least 9 months from a physicochemical point of view (Table 3). From a functional point of view, as assessed in the hydrogen peroxide challenge studies of the combination products, the performed assays did not reveal significant differences between the time of API lyophilization and following 9 months of storage at 4 °C (Figure 1, Figure 2 and Figure 3). Overall, it could be stated that the tenocyte whole cell lyophilized APIs were characterized as stable within the limits of the experimental settings. This aspect presents tangible technical and logistical advantages, as an off-the-shelf API or product presentation may be considered, allowing for an on-demand availability and the potential for widespread and facilitated material distribution.

With regard to the protein contents of the bulk cellular materials, it could be assessed that relatively low quantities of specific proteins were conditioned in each API vial for lyophilization, based on the multiple dilution factors applied during the processing of the bulk cellular materials for filling (Table 1, Tables S4 and S5). Specifically, it was determined that the theoretical quantities of the most abundant and detected proteins (e.g., MMP-2, TIMP-2, sEGFR) in the final form of the API would not individually exceed the relatively low quantitative value of 2.5 ng per dose unit (i.e., corresponding to 1.5 × 10^6^ cell unit equivalents/vial, Table 1). Therefore, considering that the EC_50_ values for the identified proteins (i.e., where applicable) are generally expressed in µg/mL, it could be stated that the considered APIs are unlikely to exert relevant therapeutic principal or ancillary effects of pharmacological or metabolic nature, provided the relatively low quantities of the individual proteins and factors (Table 1). While some additive or synergistic effects of the complex combinations of said proteins and factors cannot be ruled out, the resulting potency of said effects from a pharmacologic or metabolic viewpoint can probably not be compared in terms of scale to the potency of the antioxidant effects determined herein for the lyophilized APIs (Figure 4).

To further illustrate the differences in scale between the detected quantities of proteins in the studied tenocyte-derived lyophilized APIs and the reported therapeutic doses of the same proteins, selected publications in the field of regenerative medicine presenting the use of individual growth factors were analyzed [50,51,52,53]. Akita et al. have reported and discussed the use of FGF-2 at doses of 100 µg/mL in wound healing applications, which correspond to the approved growth factor concentration for skin ulcers in human patients [50]. Zhang et al. have reported the use of tendon stem cell (i.e., stimulated with 10–80 ng/mL HGF) conditioned medium for the healing promotion of injured Achilles tendons [51]. Hagerott et al. have described the treatment of murine dermal wounds using topical FGF-1 in quantities of 0.6–6.0 µg/cm^2^ [52]. Finally, Galiano et al. have described the topical use of 20 μg of VEGF every other day for five doses on skin full thickness wounds in genetically diabetic mice [53]. In the proteomic results of the presented study, the bulk cellular materials used for the API manufacture step were determined to contain (i.e., in API quantities corresponding to a single unit dose of 1.5 × 10^6^ cell units/vial) 810 pg of FGF-2, 527 pg of HGF, 44 pg of FGF-1, and 19 pg of VEGF-A, respectively (Table 1). While the regenerative processes of skin and of tendon tissues are different and may not be directly compared, the significant differences in the scale of detected protein quantities in the lyophilized tenocyte-derived APIs as compared to the reported therapeutically useful growth factor doses could be noted (i.e., difference factor values of 20 to 10^6^ for the growth factors discussed herein). These considerations further enable to state that, while numerous growth factors and proteins are present in the considered APIs, the contributions thereof to the intended exerted effects of a potential therapeutic product are in all probability not of pharmacological or of metabolic nature.

### 4.2. Lyophilized Progenitor Tenocyte Derivative APIs Robustly Enhance HA Rheological Behavior and Stability in Hydrogen Peroxide Challenge Assays

The reported results of this study on the HA hydrogel rheological behavior modulation properties of the selected progenitor cell derivative APIs have been assessed as robust, due to the consistent nature of the obtained effects in the various investigated experimental settings (Figure 1, Figure 2 and Figure 3). Furthermore, the use of relatively elevated experimental doses of hydrogen peroxide was useful in the accelerated degradation assays for the demonstration of the significant and differential effects of the various formulation compositions on the resulting product viscoelastic behavior in vitro. However, it is noteworthy that such hydrogen peroxide doses (i.e., 2–6% in assay samples) are far superior to the physiological quantities of products yielding reactive oxygen species in vivo, even when considering inflamed tendon tissues for example. This aspect may be interpreted positively herein and would indicate that the presented significant effects of the enhanced resistance to ROS-mediated degradation procured by the HA-API combinations are conservative, as the experimental settings were designed as more extreme than the worst-case in vivo scenario.

Due to the presence of saccharose in the cellular derivative lyophilizates (i.e., primarily included as an API lyo-protectant) and considering the H_2_O_2_ challenge assay results of the placebo formulation group, a part of the enhanced resistance of the API-loaded hydrogels to the oxidative degradation is in all probability due to antioxidant (i.e., non-detectable in the Trolox assays) and protective effects (e.g., potential water-structuring molecular action) of this carbohydrate, which have been previously described (Figure 1, Figure 2 and Figure 4) [23,54]. Furthermore, as the endpoint rheological product behaviors (i.e., expressed as the viscoelasticity parameter values) of the formulations containing the cellular derivative APIs were systematically assessed as superior as compared to the placebo and HA control groups (i.e., although not always significantly) in the various experimental conditions, it is probable that an additive or synergistic effect of antioxidant protection and of viscosity modification is procured by the inclusion of said APIs (Figure 1, Figure 2, Figure 3 and Figure 4, Tables S2 and S3). By definition, the complex modulus or complex viscosity is a measurement of the total resistance of the assay material to deformation, which may have twofold contributions: (i) an elastic contribution measured by G’ and (ii) an inelastic or viscous contribution dissipated as heat, measured by G”. The fact that the addition of the APIs increased the complex viscosity of the considered product formulas probably resulted from an increased resistance to deformation, itself due to an increased fluid viscosity and to the antioxidant API effects preserving the HA hydrogel polymer chain lengths, and thus its elastic capacity G’. This was observed as being specifically partly the case following the initial transient rise in the formulation viscoelasticity values in the 10% *w*/*w* hydrogen peroxide challenge assays (Figure 2). Specifically, it appeared that said transient effects were due to the presence of the cellular derivatives in the samples, as the placebo formulations did not present such behaviors with regard to the storage (G’) and the loss moduli (G’’) or to the complex viscosity (η*, Figure 2).

Of note, the experimental results presented in Figure 2 showed that the inclusion of the selected APIs (i.e., lyophilized progenitor tenocyte-derived whole cells or cell lysates) in the hydrogels leaded to significant modifications of the initial rheological properties of the system (i.e., pre-challenge), as compared to the HA control and to the HA-placebo groups, respectively, but without significant differences between the respective results of both API groups (Figure 2). This aspect indicated that the APIs exerted an effect which was measurable and significant on the considered rheological parameters of the system, but said effect was independent from the maintenance or the loss of the structural integrity of the cells in the APIs (i.e., due to differential manufacturing processing of the bulk cellular materials). Similarly, the results of the intrinsic antioxidant activity assessment presented in Figure 4 indicated that the effects of the APIs were significant and may be attributed to the cellular components present in the APIs (i.e., almost no intrinsic antioxidant activity of the placebo formulations). With no significant differences found between the respective results of both API groups, it was also confirmed that the intrinsic antioxidant effects were independent from the maintenance or the loss of the structural integrity of the cells in the APIs (Figure 4).

Therefore, it could be overall stated that the specific effects of the considered APIs were conservative in both the experimental setups used for the assessment (i.e., pre-challenge timepoint in rheology studies and intrinsic antioxidant activity measurement), despite the differential manufacturing processing of the bulk cellular materials included in the APIs (i.e., lyophilized whole cells or preparation of cell lysates before lyophilization) (Figure 2 and Figure 4). The exact reason for the independence of the API effects from the manufacturing process, and thus from the final structural parameters of the cellular materials included in the lyophilizates, remains to be elucidated. The observed effects were due to the presence of the API particles (i.e., whole cells and fractions of cells), and seemed independent from the size distribution parameters of said particles (i.e., different size distributions of the included particles, as presented in Figure 5). It could be postulated that for the antioxidant activities of the APIs, conserved between both API groups, the activity was due mainly to biological components readily accessible in both groups (i.e., either provided by exposed external membrane constituents or by internal protein structures, made available in the whole cell API group by permeation of the cellular membranes during lyophilization) (Figure 4). In the pre-challenge context of the accelerated degradation assays presented in Figure 2, it could be hypothesized that both forms of the cellular components of the APIs (i.e., whole cells and cell lysates) interacted with the HA polymeric system in a similar way (e.g., modulation of the water-structuring activities of HA) by physical action or by complex chemical interactions or bonding with the polysaccharidic chains. However, the fact that in the same experimental setup and during the subsequent endpoint rheological analysis after the challenge, the whole-cell APIs procured systematically higher rheological values tended to indicate that the conserved cellular structures in the APIs procured some advantages against ROS-mediated degradation. Therein, the existence of defined, although permeated, cellular bodies potentially provided enhanced functionality as regards ROS scavenging or ROS sequestration.

Furthermore, the exact mechanism leading to the reported transient initial surge in the hydrogel viscosity as visualized in Figure 2 remains unclear but might be differentially or cumulatively explained by the potential generation of carbon dioxide upon biological material reaction with the hydrogen peroxide, by changes in the system microstructure upon initiation of the deformation, by relative swelling of the polymers or of macromolecular structures submitted to oxidative stress, or by facilitated chemical interactions between biological materials and the HA chains in the highly oxidative environment (Figure 2). For the whole cell derivative API group, inclusion of the particles in relatively high quantities in the hydrogel (i.e., 10^6^ equivalent cell units/mL) would constitute a possible explanation for the relatively enhanced viscoelasticity values, however the transient nature of the observed effect and the fact that cell lysates produced comparable effects argue against this theory (Figure 2). It could also be stated that the inclusion of an oxidant agent in the system was necessary for the observation of the transient relative rise in the rheological property values, a resultant of the various potential mechanisms described hereabove.

Overall, the observed important initial viscosity modification effects in the accelerated oxidative product degradation assays were in all probability due to the artificial and extreme parameters of the in vitro experiments and thus yield low impact on the further product development approaches and on the eventual clinical applications, as the same levels of oxidative stress will not realistically be encountered in vivo. Furthermore, the main advantages of the presented combination products reside in the relative terminal increase in the key rheological parameter values, which constitutes an indirect endpoint marker of hydrogel resistance to the oxidative degradation (Figure 2 and Figure 3).

### 4.3. Designing of HA-Progenitor Cell Derivative Combination Products Yielding Intrinsic Antioxidant Properties Leads to the Enhancement of Functional and Stability Parameters

While the use of stabilized HA-based hydrogels has been extensively described for the delivery of therapeutic APIs including biologicals, published reports on the converse or complementary use of cells or cell derivatives for hydrogel stability enhancement remain scarce [55]. Diverse approaches to hydrogel viscosity/viscoelasticity modulation (e.g., chemical modification, inclusion of thermoresponsive polymers) have been reported for enhancement of the functional and of the therapeutic characteristics of the product or for the facilitation of product processing [56,57,58]. Aside from meeting of specific functional requirements, tuning of the HA-based hydrogel rheological behavior has been an objective or a tool of product stability enhancement, namely for protection against enzymatic and oxidative degradation [59]. In this context, the specific modulation of the intrinsic antioxidant activity of HA has been achieved in various ways, notably with the inclusion of protective carbohydrates, which may also act as effective lyo-protective agents [60,61]. Furthermore, the need for standardized assessment of the correlation between the in vitro rheological properties of HA-based hydrogels and the relevant clinical parameters of the products has been established and described for dermal fillers but may be extrapolated to alternative HA formulations for different therapeutic uses [62,63].

Overall, it may be stated that the considered lyophilized progenitor tenocyte derivative APIs procure multiple specific benefits when extemporaneously reconstituted in the appropriate HA-based hydrogels. Firstly, the function of saccharose as a lyo-protectant (i.e., final proportion of 8.0% *m*/*v* saccharose in the lyophilization mix) is commuted to a visco-protective effect following the API reconstitution in the HA hydrogel (i.e., final proportion of 4.0% *m*/*v* saccharose in the considered hydrogels). Secondly, the presence of cellular derivative APIs possessing intrinsic antioxidant properties further contributes to both the internal protection of the therapeutic product and to the potential ROS scavenging activities after the in vivo administration in wounded tissues, by extrapolation of the characterized mechanisms of action of cell therapies (Figure 4) [45]. Thirdly, the inclusion of whole cell structures or of sub-cellular vesicular bodies in therapeutic products potentially provides enhanced tissue gliding properties, which may be of interest for tendinous tissue affections [47]. Fourthly, the administration of exogenous extracellular matrix (ECM) components, albeit in relatively low overall quantities, may be beneficial from a mechanical standpoint as well as within the orchestration of tissue regeneration or repair mechanisms. Specifically, previously reported proteomic characterization results of FE002-Ten progenitor tenocyte bulk cellular materials had identified the main ECM constituents (e.g., collagens, fibronectin, GAGs, elastin) as relatively abundant in terms of quantity [64].

Specifically, it is probable that a multifactorial composite effect results from the combination of the various constituents of the considered API formulas. Such an effect may be relatively complex to characterize and will in all probability differ depending on the experimental setup (i.e., in vitro biomechanical or cellular assays versus in vivo preclinical experiments). Notably, the determination of the specific contributions of each effect parameter, and in particular the relative weight pondering each parameter within the overall claimed mechanism of action or exerted effect will yield determining information relative to the regulatory classification of the considered combination products.

Of note and in addition to the technical advantages of formulation stability enhancement procured by the various components of the considered therapeutic combination products, potential clinical advantages may be foreseen as follows. Tendinopathies have been characterized notably by the presence of local tissular inflammation and highly oxidative states, which may be damaging but also represent a useful component of the evolutive healing phases of such musculoskeletal tissues [65,66,67]. The interest of administering a product with potential multi-facetted antioxidant activities (i.e., intrinsic or ancillary antioxidant properties of APIs, HA, and saccharose) may be twofold, with a potential direct influence on the product implantation environment (e.g., ROS scavenging activities) and converse in situ stimulation of the implanted product by the recipient’s pathological (i.e., increased oxidative) tissular environment. Indeed, it was shown herein that the considered combination products required the presence of an oxidant for the optimal modulation of the hydrogel viscoelastic behavior, as such rheological property variations were observed in the H_2_O_2_ degradation assays, yet the combination products presented identical injectability profiles in vitro in the absence of an oxidative challenge (Figure 1, Figure 2 and Figure 6). Therefore, the inflammation state naturally present in tendon tissue lesions themselves may be harnessed and may indirectly benefit the therapeutic process mediated by the considered therapeutic products, by potentiating and enhancing hydrogel stability parameters and therefore enhancing the primary product function (i.e., in terms of effect amplitude or effect duration).

### 4.4. Progenitor Tenocyte Derivative APIs and HA Combination Products Present Several Technical Advantages

Use of hyaluronic acid as a therapeutic active pharmaceutical ingredient or as a functional delivery vehicle presents numerous advantages, among which the extensive available in vitro and clinical data, an excellent biocompatibility, and a large industrial hindsight around raw material sourcing and product manufacturing [68,69,70]. Along with its natural presence in human tissues, high biocompatibility and safety characterize HA-based formulas, which have been notably used for treating tendinopathies or burn wounds and were recently considered for various inhalation applications [7,71,72]. On the other hand, inclusion of progenitor tenocyte derivative APIs in therapeutic products or medical devices is technically enabled from a safety and quality point of view by the extensive traceability, testing, and qualification steps performed during the cell sourcing phase and during subsequent GMP cell banking [44]. Inherent advantages of specifically sourced primary progenitor cell types comprise a defined and consistent tissue-specific phenotype, a high expansion potential in vitro, low probability of immunogenicity and tumorigenicity, and high stability in storage [42]. Multi-tiered progenitor cell banking further enables highly sustainable cellular starting material and bulk material provision, along with sufficient retention samples for iterative quality controls.

While specific progenitor tenocyte sources have been preliminarily qualified in preclinical settings for allogeneic homologous cell therapy development, applications in tendinopathies suffer from a widespread problematic encountered in the general cytotherapy field, namely the extremely low cell engraftment or low persistence of the delivered cellular payloads [47]. Indeed, the inflammatory environment of affected tendons coupled to the high mechanical stress exerted in and around the tissues of interest probably do not favor cell viability or integrity maintenance after the product application. Such considerations may therefore serve as a rationale for the development of cellular derivative-based products, which may retain the appropriate functions while yielding relative technical, regulatory, and safety advantages [48].

Manufacturing considerations around the conjoint use of linear HA and specific banked primary progenitor cells (e.g., FE002-Ten cell source) additionally highlight the relative simplicity and the high robustness of both respective component types. Indeed, linear HA may be simply produced in adequate clinical grades and quantities through modern biotechnological platforms [8,18]. Similarly, primary progenitor tenocytes are culture-expanded in standard adherent in vitro incubation conditions which require minimal manipulations and simple processing (i.e., CO_2_ incubation, use of FBS medium supplements). Subsequent downstream processing using physical processes (e.g., thermal cell lysis, lyophilization) may be precisely controlled and adapted to individual manufacturing requirements. Overall, such aspects governing the raw material or starting material processing allow for optimal devising of risk-based and quality-driven approaches to workflow and product development [48].

Specifically, appropriate preservation of polymers or of cellular derivative-based APIs by lyophilization potentially enable tangible wide and early access to combination products, with the obtention of stable off-the-shelf formulations and independence from mandatory cold-chain transport and storage, as mentioned previously. These major logistical and economic gains may be quantified for such products, by comparison to the classical workflows of cell therapy preparation and administration, which require lengthy manufacturing delays and cumbersome cryopreservation storage [49]. Overall, such bottleneck phases and parameters drastically restrict the number of patients potentially eligible for treatment with considered classical cell therapies. Therefore, the ability to obtain stable combination products requiring minimal logistical resources (e.g., refrigerated storage and extemporaneous aseptic reconstitution) is of high interest for supplying potential cell-derived cell-free therapeutic products or medical devices for managing specific tendinous tissue affections. On a manufacturing note, while the search continues for the best manufacturing process or technique to stabilize cell-based APIs (i.e., methods potentially requiring less energy or presenting better ecological sustainability), lyophilization is applied as it currently benefits from proven effectiveness, high public interest, extensive documentation, and industrial hindsight.

Particular importance for combination product conditioning is set on the final administration system (i.e., appropriate syringe), which should be designed to facilitate the manipulation and the administration by the physician. It was demonstrated herein in vitro and ex vivo that the considered product formulas presented good syringeability and injectability parameters (Figure 6 and Figure S10). Specifically, the retained administration system was chosen to be as close as possible to standard clinical practice in tendinopathy management (i.e., small bore needle, injection volumes < 2 mL), while being sufficiently modulable for ease of manufacturing scale transposition. With mean required injection forces < 50 N for all the considered product formulas in the equine tendon ex vivo setting, it was assessed that the considered systems presented good syringeability, as defined and discussed by Cilurzo et al. [73]. Specifically, with regard to the relatively constant required injection forces during the extrusion of the products, it did not appear that the APIs caused a particle build-up or plugged the relatively small-bore needle (Figure 6 and Figure S10).

### 4.5. Progenitor Tenocyte Derivative APIs and HA Combination Products Possess Several Potential Therapeutic Applications

Despite the high incidence and prevalence of tendon tissue traumatic or degenerative affections, few approved therapeutic means allow for rapid and effective restoration of both tissular structure and function [65,74]. Apart from symptomatic management, various potential curative therapies or products have been proposed, notably around the use of autologous PRP and the administration of allogeneic stems cells or stem cell derivatives [66,67,75]. While several studies have outlined potential benefits in preclinical models of tendon affection management, limited conclusive evidence has been submitted to date around the clinical use and around the efficacy of stem cells and derived cell-free therapeutic products [76,77,78,79,80,81]. In autologous settings, the largest reported evidence covers the therapeutic use of PRP, yet outcomes are described as being highly dependent on the indication and on the retained treatment protocol [82]. When considering acellular formulations for tendinopathy indirect or direct management, HA-based hydrogels have yielded strong evidence of beneficial effects, which could be explained by tissue lubrication or gliding enhancement, immunomodulatory properties, and intrinsic anti-inflammatory properties [83,84,85].

Based on existing therapeutic products and on published studies for tendinopathy management, the considered combination products incorporating HA and progenitor cell derivative APIs may potentially be of use in specific post-traumatic interventions or in tissue degenerative affections [11,12,13]. Specifically, while volumetric tissue losses often require allogeneic/xenogeneic tissue or synthetic construct grafting, complementary treatment with localized hydrogel product injections may be helpful to avoid adhesions or to favor the graft integration and the functional restoration. Furthermore, the early management of inflammatory or degenerative tendon disease states may constitute alternative indications of such complex products, potentially in replacement of corticosteroid infiltrations or in parallel to various forms of physical treatments [47]. Generally, depending on the proposed mechanism of action and the intended effects to be procured by the product, the exact therapeutic window may vary when considering different tendinopathy types and affected anatomical sites (Figure S5). Therein, specific care should be taken in the adequate matching of tissular needs and provided therapeutic effects, at the appropriate respective phases of physiological tendon healing.

Although many of the presented assays in this study for the rheological characterization of products were performed using 1% *w*/*v* HA with a molecular weight of 1.2–1.5 MDa, the results have outlined that the observed effects of viscosity modification of the cellular derivative APIs are robust and consistent in nature for the various hydrogel formulas (i.e., different HA concentrations and HA MW, Figure 3). Therefore, appropriate formulation and processing parameters of the hydrogel base of the combination product prototypes may be considered for applications in tendon affection management, wherein commercialized products usually contain a higher HA concentration or a higher MW for the HA hydrogel base polymer (e.g., Ostenil^®^ Tendon (2% HA, 1.6 MDa, with 0.5% mannitol), Orthovisc^®^-T (1.5% HA, 1.0–2.9 MDa), or Suvenyl^®^ (1% HA, 2.7 MDa)) [11,86].

### 4.6. Regulatory Considerations Orient the Development of Progenitor Tenocyte Derivative APIs and HA Combination Products toward a Class III Medical Device

From a regulatory standpoint, developing cell therapies using cultured and viable primary progenitor tenocytes delivered in an HA vehicle would automatically fall under the category of combined advanced therapy medicinal products (cATMP) in Europe and would be defined as a standardized transplant product (TrSt) in Switzerland [47]. Due to several factors such as insufficient therapeutic cell engraftment, elevated development costs, and the previously mentioned logistical challenges of classical cytotherapies, alternative technical development and related product classification procedures may be of interest for the design of novel cell derivative-based products for the management of tendinopathies. Therefore, appropriately processed active substances and APIs could potentially be included in medical devices (MD), wherein the validity of such classification depends on the definition of the primary/principal and of the ancillary mechanisms of action of the product [87]. With many approved uses in various product categories, HA is a prime example of sometimes borderline product regulatory classification or authorization [88].

Specifically, while MD primary mechanisms of action may not be pharmacological, immunological, or metabolic in nature in European legislation (EU-MDR), ancillary mechanisms of action may be of such nature, for an enhanced obtention of the claimed therapeutic effect [87]. For tangible consideration of such classification, key elements of functional qualification, therapeutic indication definition, and manufacturing process specification constitute some of the prerequisites for the proposed progenitor tenocyte derivative API-HA hydrogel combination products. While various steps may be undertaken for rationalization of risks, costs, and length of the product development, the evolutive regulatory landscapes prompt careful consideration and forward planning during core technology and product development, for optimal insurance of sustained regulatory compliance throughout the product lifecycles.

Considering the proposed combination product formulas for potential application in tendinopathies, it may be assessed that the principal mode of action (i.e., defined as necessary and sufficient) is fulfilled by the presence of HA as a hydrogel base, which will mainly procure lubrication and gliding enhancement effects, as well as mechanical reduction of friction and pain. Such considerations have notably been the bases for registration of the above-mentioned products as MDs for the management of tendinopathies. Additionally, lyo-protective API constituents such as selected carbohydrates may exert an ancillary effect in the reconstituted combination product, through antioxidant protection of the HA polymer and through hydrogel viscosity modulation. Such antioxidant effects are further potentiated or supplemented by the intrinsic antioxidant capacities of the considered primary progenitor tenocyte derivative APIs (Figure 4). Finally, while it is not completely excluded (i.e., for dose reasons) that specific cellular derivative APIs may partly act through pharmacologic or metabolic means, these effects in all probability remain ancillary to the viscosity modulating effects exerted on the hydrogel in specific oxidative settings, as well as the therapeutic delivery of ECM components or vesicular biological constituents. Overall, while the proposed combination products may possess multifactorial complex effects, the dominating component of said effects remains that of HA, which serves as the basis for tentative consideration of the proposed formulas under applicable MD regulations. Therefore, provided that such considerations are in adequation with current national or supranational laws and regulations, further development work could be performed in view of registering class III MDs (i.e., implanted, defined as high-risk in ad hoc classifications), based on combinations of progenitor tenocyte derivative APIs and HA hydrogels, in selected countries for the management of tendinopathies.

## 5. Conclusions

The purpose of the present study was the experimental in vitro characterization of selected product formulas incorporating progenitor tenocyte-derived APIs in HA-based hydrogels, in view of developing potential optimized combination products for the therapeutic management of tendon tissue disorders in the form of medical devices. Physicochemical and functional characterization of the lyophilized cell-derived APIs revealed relatively low protein contents, high lyophilizate stability, and significant intrinsic antioxidant properties of the APIs. Stability enhancement of HA-based hydrogels by specified progenitor tenocyte derivative APIs was described and discussed, notably from technical, therapeutic, and regulatory points of view. Specific rheological characterization of various product formulations after the hydrogen peroxide challenge outlined significantly improved viscoelasticity values (i.e., G’, G’’, and η*) in various settings and at multiple timepoints, as compared to the HA control groups. Finally, optimal syringeability and injectability of the considered product formulas were determined in vitro and ex vivo in clinically relevant administration systems for tendinopathy management. Overall, the present study provides the technical bases for the further industrial development of progenitor tenocyte derivative-based injectable hydrogel products or devices, to potentially be therapeutically applied in tendinous tissue disorders or for adjuvant post-surgical use.

## Figures and Tables

**Table 1:** Proteomic characterization results of the bulk cellular materials (i.e., FE002-Ten primary progenitor tenocyte lysate) used for lyophilized API manufacture. The 20 most abundant proteins ^1^ were reported hereunder, in a decreasing order of appearance as classified by the relative detected quantities, along with the theoretical protein molecular weights and the corresponding normalized detected protein quantities. The calculated protein quantities in API unitary doses were listed for each entry. The relative protein quantity detected and normalized to the unfractionated cell lysate total protein content was expressed in pg of the specified protein per mg of the whole cell lysate total protein content. The calculated quantity of proteins in an API unitary dose (i.e., corresponding to 1.5 × 10^6^ cell unit equivalents) was expressed in pg of the specified protein per lyophilizate vial. API, active pharmaceutical ingredient; Da, Daltons.

Protein Abbreviated Name (Protein Full Name)	Theoretical Protein Molecular Weight ^2^ (Da)	Normalized Relative Protein Quantity in the Cell Lysate (pg/mg)	Calculated Protein Quantity in an API Unitary Dose (pg/vial)
MMP-2 (72 kDa type IV collagenase)	72,000	8504	2412
TIMP-2 (Metalloproteinase inhibitor 2)	21,000	8087	2294
sEGFR (Soluble epidermal growth factor receptor)	110,000	6807	1931
TIMP-1 (Metalloproteinase inhibitor 1)	28,000	4155	1179
sgp130 (Soluble gp130)	100,000	3751	1064
FGF-2 (Fibroblast growth factor 2)	18,000	2856	810
HGF (Hepatocyte growth factor)	83,100	1859	527
sTNFRI (Soluble tumour necrosis factor receptor type I)	18,300	743	211
MMP-13 (Collagenase 3)	54,000	737	209
IL-1Ra (Interleukin-1 receptor antagonist protein)	17,300	383	109
FST (Follistatin)	38,000	308	87
MMP-7 (Matrilysin)	28,000	190	54
FGF-1 (Fibroblast growth factor 1)	15,500	155	44
IL-23 (Interleukin-23)	55,000	115	33
ENG (Endoglin)	64,000	102	29
MDC/CCL22 (C-C motif chemokine 22)	7800	75	21
Flt-3L (Fms-related tyrosine kinase 3 ligand)	20,000	73	21
VEGF-A (Vascular endothelial growth factor A)	27,000	66	19
MCP-1 (C-C motif chemokine 2)	8700	54	15
sIL-6R (Soluble IL-6 receptor)	42,250	48	14

^1^ Other proteins which were detected in the samples by the multiplex proteomic analyses were listed in Table S5. ^2^ The theoretical protein molecular weights were determined in silico based on protein structure projections.

**Table 2:** Quantitative and semi-quantitative results of the descriptive analyses, the selected characterization testing, and the corresponding gradings for the obtained progenitor tenocyte lyophilizates, performed 24 h after the lyophilization processing. The results indicated that both of the considered API formulations and the related processing conformed satisfactorily to the predefined specifications with regard to the target parameters and the defined acceptance criteria. The semi-quantitative grading was performed by two experienced operators using the abbreviated nomenclature presented hereafter. (–) = unsatisfactory, (+) = sub-optimal, (++) = satisfactory, (+++) = optimal. Photographic records of the API lyophilizates are presented in Figure S6. The measurements were performed in triplicate unless otherwise stated. API, active pharmaceutical ingredient; NA, non-applicable; Ph. Eur., European pharmacopoeia; RH, relative humidity.

Parameters	Targets	Acceptance Criteria (*Cumulative*)	Results/Grading of the Lyophilizates		
			Placebo Formula	Cell Lysate API	Whole Cell API
Presence of cake	Presence of a solid cake	Presence of a solid cake No residual liquid phase	+++	+++	+++
Batch uniformity	Uniform lyophilizate batch	Vial-to-vial uniform aspect Dry product unitary mass uniformity ^1^	+++	+++	+++
Cake color	White cake color	White cake coloration Monochrome cake Consistent hue, tint, tone, and shade of the cake	+++	++	+++
Cake structure	Uniform structure	Presence of a single cylindrical solid mass	+++	++	++
Cake density	Dense cake	Presence of small cake pores Absence of gross porosity on the sides and bottom of the cake	+++	+++	+++
Cake finish	Shiny or sheen finish ^2^	Shiny or sheen finish observed on the top, sides, and bottom of the cake	+++	++	++
Cake friability	Non-friable cake ^3^	No detachment or detachment of small fragments from the quoins of the cake Free fragments <5% of total cake volume	+++	+++	+++
Cake topography	Consistent cake topography	Consistent presence of top flakes, bumps, cracks, concavity, or peaks	+++	+++	+++
Cake shrinkage	Minimal cake shrinkage	No horizonal shrinkage Vertical shrinkage <10% from original fill height	+++	+++	+++
Cake collapse/ meltback	No cake collapse or meltback	Absence of cake collapse Absence of observable liquid portion of the cake	+++	+++	+++
Residual material presence	Minimal residual material presence	Minimal residual material presence on the upper rim of the cake, on vial surface at the original fill height	+++	+++	+++
Particle presence	Absence of observable contaminating particles	Absence of observable contaminating particles ^4^	+++	+++	+++
Residual moisture level	Residual moisture level <5.0% ^5^ water	Residual moisture level < 5.0% water	4.0% ± 0.2%	4.2% ± 0.4%	4.4% ± 0.3%
Cake reconstitution time	Full cake reconstitution time <90 s ^6^	Absence of observable solid and undissolved mass after 90 s	<30 s	<30 s	<30 s
Cell structural integrity maintenance in the cake	Presence of structurally integral cells	Structural integrity confirmed microscopically and by size distribution analysis ^7^	NA	NA	+++
pH value after cake reconstitution	pH value of 7.5 ± 1.0 after reconstitution	Measured pH value comprised in the target interval	7.3 ± 0.2	7.2 ± 0.2	7.3 ± 0.1
Osmolality value after cake reconstitution	Osmolality value of 300 ± 30 mOsmol/kg	Measured osmolality value comprised in the target interval	296 mOsmol/kg ± 6 mOsmol/kg	287 mOsmol/kg ± 12 mOsmol/kg	290 mOsmol/kg ± 8 mOsmol/kg
Cellular devitalization upon cake reconstitution	Absence of viable cells	Absence of viability confirmed by staining of cells with Trypan blue	NA	NA	+++

^1^ The product mass uniformity determination was based on the Ph. Eur. method 2.9.5. “Uniformity of mass of single-dose preparations” with an acceptance level set at the mean mass ± 10%. ^2^ The products were examined under direct laboratory lighting. ^3^ The cake friability was assessed by vortexing the lyophilized samples at maximum speed for 10 s on a benchtop vortex. ^4^ Assessment of contaminating particle presence was based on the Ph. Eur. method 2.9.20. “Particulate contamination: Visible particles”. ^5^ The residual moisture levels were determined by the Karl–Fisher method. ^6^ The sample reconstitution was assessed after the addition of the adequate solvent and after gentle manual shaking of the samples. ^7^ The particle size distribution analysis was performed using Mastersizer instruments. Table adapted from Laurent et al., 2021 [48].

**Table 3:** Quantitative and semi-quantitative results of the lyophilized progenitor cell derivative API (i.e., whole cell lyophilizates) long-term stability studies. The vials were stored in the specified conditions, protected from the light following manufacture, and were equilibrated at ambient temperature overnight before the assessments and the measurements were performed. The characterization was performed using the methods described or referenced in Table 2. The descriptive parameter evaluation was performed by two experienced operators and the semi-quantitative grading was performed using the abbreviated nomenclature presented hereafter. (–) = gross deterioration of the cake, (+) = observable modifications of the cake, (++) = minimal observable modifications of the cake, (+++) = no observable modifications of the cake. The measurements were performed with *n* = 5. API, active pharmaceutical ingredient.

Storage Period	Storage Temperature	Descriptive Parameters ^1^	Endpoint Moisture Level	Endpoint Reconstitution Time	Endpoint pH Value
3 months	−20 °C	+++	3.9% ± 0.5%	45 s	7.1 ± 0.2
	4 °C	+++	4.6% ± 0.4%	35 s	7.1 ± 0.3
	22 °C	+++	4.5% ± 0.3%	35 s	7.2 ± 0.3
	37 °C	+++	4.3% ± 0.5%	50 s	7.1 ± 0.4
6 months	−20 °C	+++	4.2% ± 0.3%	45 s	7.0 ± 0.2
	4 °C	++	4.4% ± 0.3%	45 s	7.1 ± 0.1
	22 °C	+++	4.3% ± 0.5%	40 s	7.3 ± 0.2
	37 °C	+++	4.5% ± 0.4%	50 s	7.2 ± 0.3
9 months	−20 °C	+++	4.0% ± 0.1%	45 s	7.1 ± 0.3
	4 °C	+++	4.5% ± 0.5%	40 s	7.1 ± 0.3
	22 °C	++	4.4% ± 0.4%	35 s	7.2 ± 0.1
	37 °C	+++	4.5% ± 0.3%	45 s	7.2 ± 0.3

^1^ The gradings of the descriptive parameters corresponded to the addition and the semi-quantitative result ponderation of all the organoleptic and descriptive assays listed in Table 2.

## Data Availability

The data presented in this study are available on request from the corresponding author. The data are not publicly available due to legal and to statutory restrictions.

## Supplementary Materials

The following are available online at https://www.mdpi.com/article/10.3390/pharmaceutics13122196/s1. Figure S1: Multi-tiered cell banking workflow for cellular starting material production, Figure S2: Process for API starting material processing and stabilization, Figure S3: Timecourse of human progenitor tenocyte in vitro proliferation, Figure S4: Lyophilized API characterization workflow: Experimental steps, Figure S5: Potential therapeutic applications of combination products, Figure S6: Lyophilized API descriptive analysis and photographic records, Figure S7: Whole cell API microscopic characterization photographs, Figure S8: Standardized lyophilized API reconstitution workflow, Figure S9: Ex vivo tendon tissue samples: Syringeability assay setup, Figure S10: Detailed force injection profiles of combination products, Table S1: Quantitative results of lyophilized progenitor cell derivative particle size distribution analysis, Table S2: Statistical parameters of rheological profiles presented in Figure 1, Table S3: Statistical parameters of rheological profiles presented in Figure 2, Table S4: Proteomic database entries relative to the quantified API proteins listed in Table 1, Table S5: Additional proteomic data complementing the results listed in Table 1.

## Abbreviations

API	active pharmaceutical ingredient
BCA	bicinchoninic acid assay
cATMP	combined advanced therapy medicinal product
CHUV	centre hospitalier universitaire vaudois
CMV	cytomegalovirus
Da	Daltons
DMEM	Dulbecco’s modified Eagle medium
DMSO	dimethyl sulfoxide
EBV	Epstein-Barr virus
EDTA	ethylenediaminetetraacetic acid
FBS	fetal bovine serum
GAG	glycosaminoglycan
GMP	good manufacturing practices
GO	gene ontology
HA	hyaluronic acid
HBV	hepatitis B virus
HCV	hepatitis C virus
HIV	human immunodeficiency virus
H_2_O_2_	hydrogen peroxide
HSV	herpes simplex virus
HTLV	human T-cell lymphotropic virus
LVE	linear viscoelastic region
MCB	master cell bank
MD	medical device
MW	molecular weight
Pa	Pascals
Pa·s	Pascal seconds
PBS	phosphate-buffered saline
PCB	parental cell bank
PDT	population doubling time
PDV	population doubling value
Ph. Eur.	European pharmacopoeia
PRP	platelet-rich plasma
QC	quality control
ROS	reactive oxygen species
TEAC	Trolox equivalent antioxidant capacity
TrSt	standardized transplant product
USA	United States of America
UV	ultraviolet
WCB	working cell bank

## Appendix A

A schematic technical overview of the main steps of the present study is provided as a workflow in Figure A1, for the facilitated comprehension of the sourcing, manufacturing, and characterization activities around the considered APIs and the combination products, respectively.
