# Supplementary material for: Combination of Hyaluronan and Lyophilized Progenitor Cell Derivatives: Stabilization of Functional Hydrogel Products for Therapeutic Management of Tendinous Tissue Disorders
Source: Pharmaceutics. 2021 Dec 19;13(12):2196. doi: 10.3390/pharmaceutics13122196 (PMC8706504; doi:10.3390/pharmaceutics13122196)
Supplement: Supplementary file 1 [file pharmaceutics-13-02196-s001.zip › pharmaceutics-1482435-supplementary.pdf]

Supplementary Figures

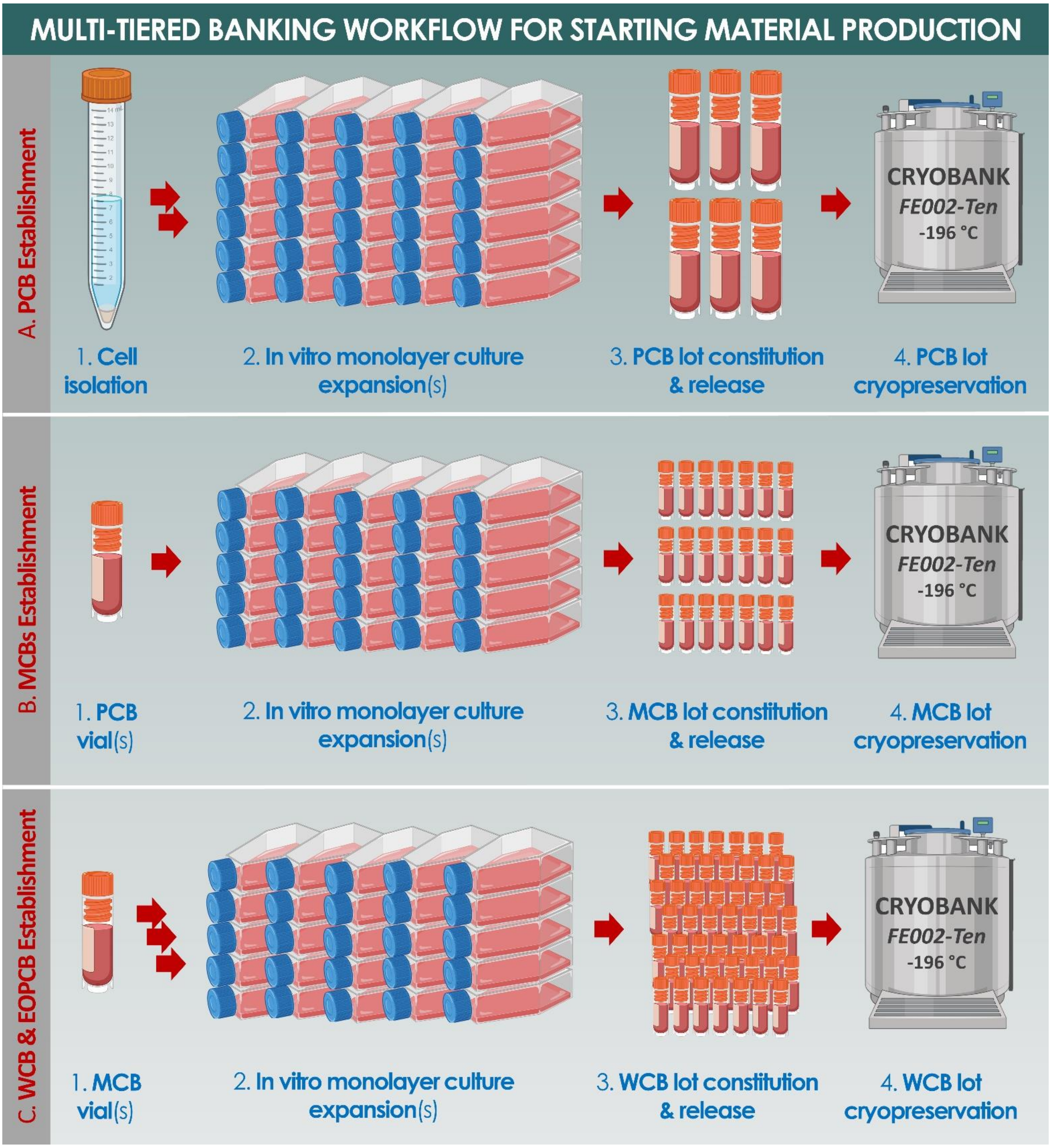

Figure S1. Schematic workflow for multi-tiered banking of human progenitor tenocytes (e.g., FE002-Ten primary cell type) to be used as lyophilized API bulk starting materials. (**A**) Following appropriate cell isolation from the original tissue donation (e.g., enzymatic cell isolation), preliminary in vitro culture-expansions are performed to establish the parental cell bank (PCB) at low passage levels (e.g., passage levels 1–2). (**B**) PCB materials are used for further in vitro culture-expansion of the primary progenitor cells, in order to establish the master cell bank (MCB) at intermediate passage levels (e.g., passage levels 3–4). (**C**) MCB materials are used for further in vitro culture-expansion of the primary progenitor cells, in order to establish the working cell banks (WCBs) at pre-production passage levels (e.g., passage levels 5–8). The WCB materials are subsequently further serially expanded for the constitution and the qualification of end of production cell banks (EOPCBs, established at passage levels 1.5 × higher in value than the highest WCB passage level). API, active pharmaceutical ingredients; EOPCB, end of production cell bank; MCB, master cell bank; PCB, parental cell bank; WCB, working cell bank.

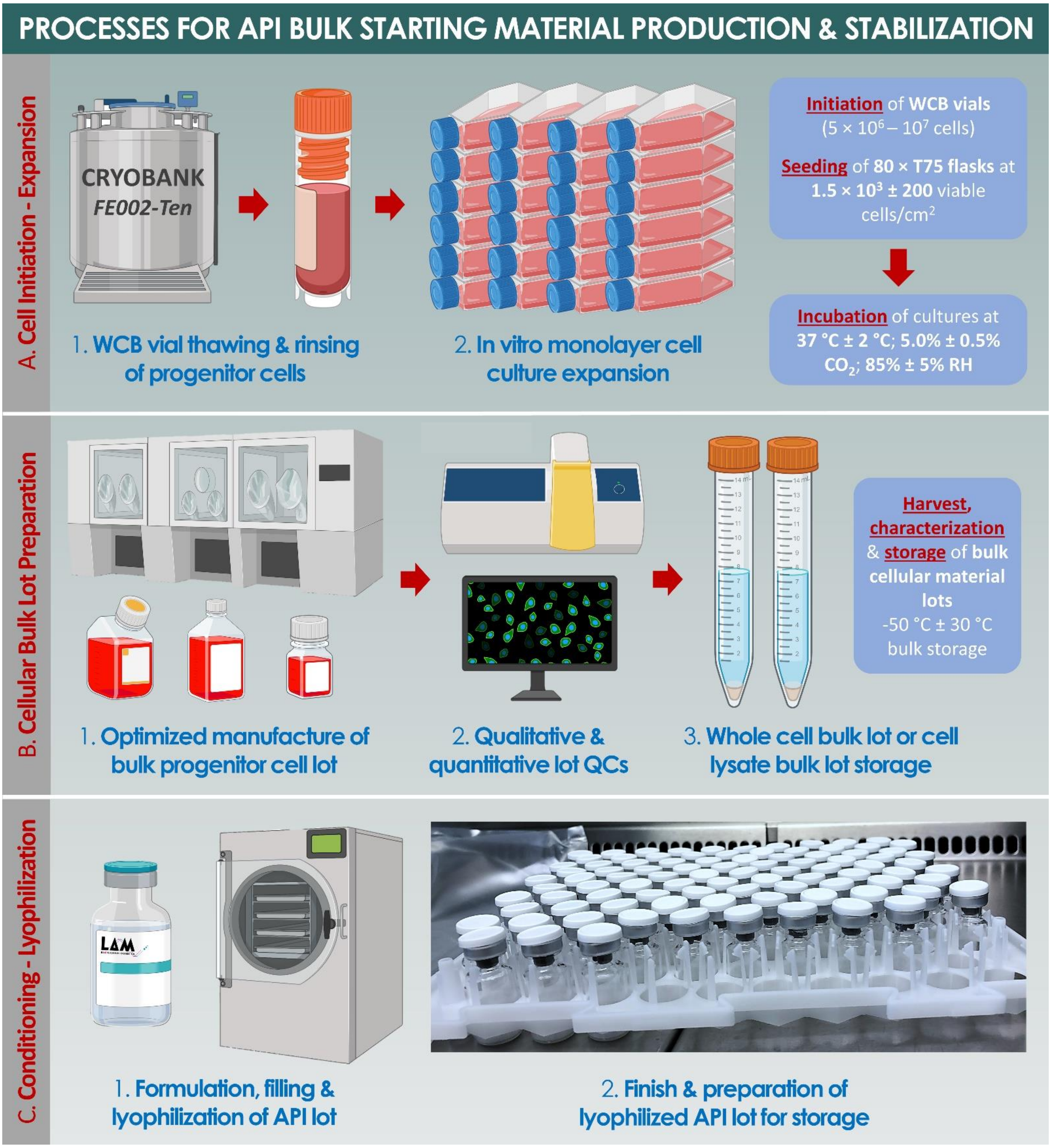

Figure S2. Processes for lyophilized progenitor tenocyte API bulk starting material processing and stabilization. (**A**) Progenitor cell WCB vials (e.g., FE002-Ten cell type) are initiated from liquid nitrogen storage and the cells are culture-expanded as appropriate for generation of a bulk cellular material lot. (**B**) Following the defined technical specifications and using optimized conditions and materials, the bulk cellular material lot (i.e., whole cells or cell lysates) is harvested, characterized, and conditioned for storage. Generation of cell lysates requires an additional phase of thermal structural disruption of the harvested cells. (**C**) After appropriate formulation of the bulk cellular materials with cryo- and lyo-protectants, the suspensions are conditioned in individual glass vials and are processed for lyophilization. Subsequently, lyophilized API lots are characterized and qualified before release and storage. API, active pharmaceutical ingredient; QC, quality control; RH, relative humidity; WCB, working cell bank.

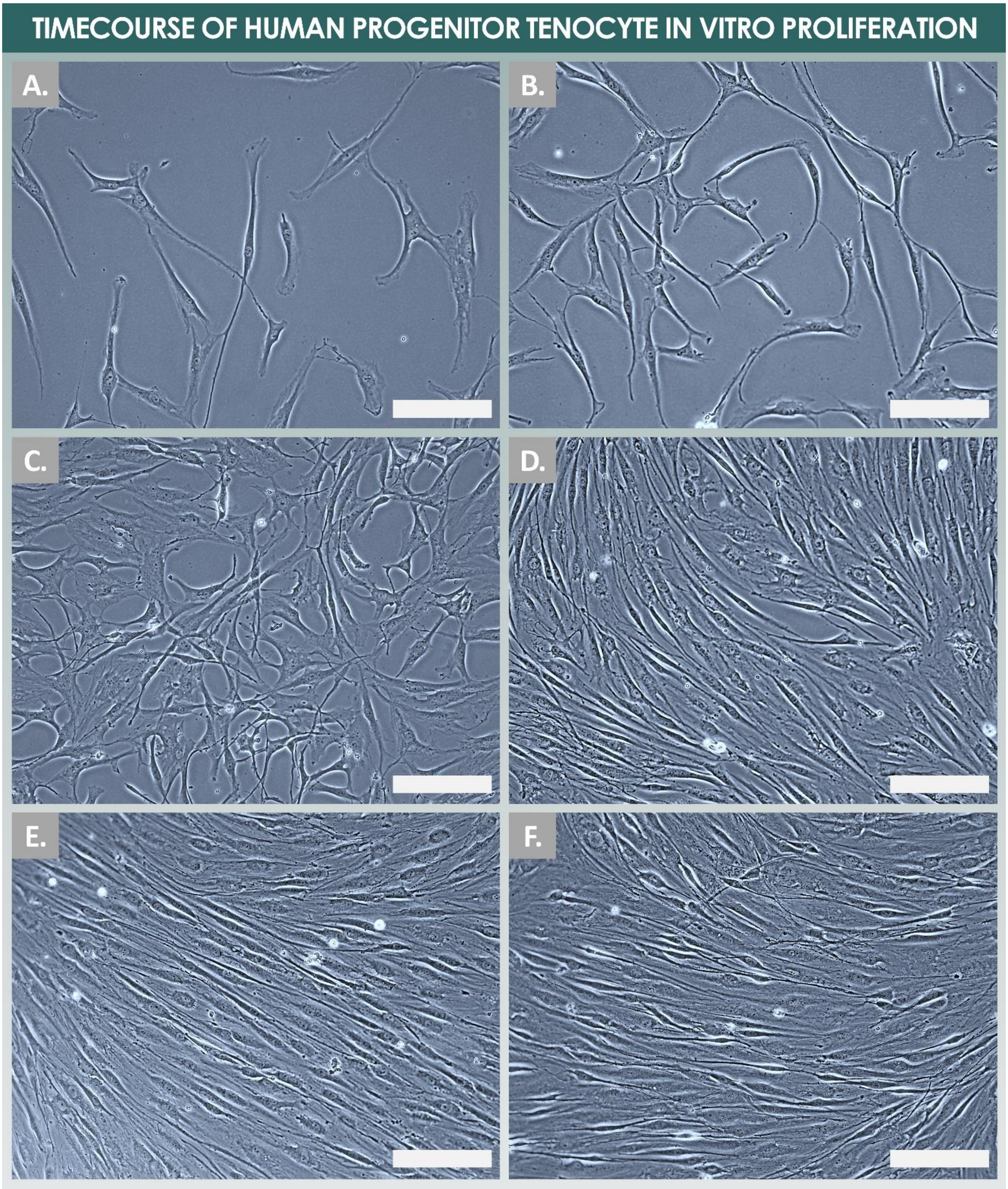

Figure S3. Timecourse of human primary progenitor tenocyte (e.g., FE002-Ten primary cell type) in vitro monolayer expansion. The cells were seeded in T75 cell culture flasks at passage level 6 and the presented photographic records were obtained iteratively at the time of the medium exchange procedures under contrast phase microscope visualization. (**A**) Proliferating cells after 2 days of culture. (**B**) Proliferating cells after 5 days of culture. (**C**) Proliferating cells after 7 days of culture. (**D**) Proliferating cells after 9 days of culture. (**E**) Proliferating cells after 12 days of culture. (**F**) Proliferating cells after 14 days of culture. Subsequently, the cells were harvested and were further processed on the 15th day following the culture initiation step. Scale bars = 75 μm.

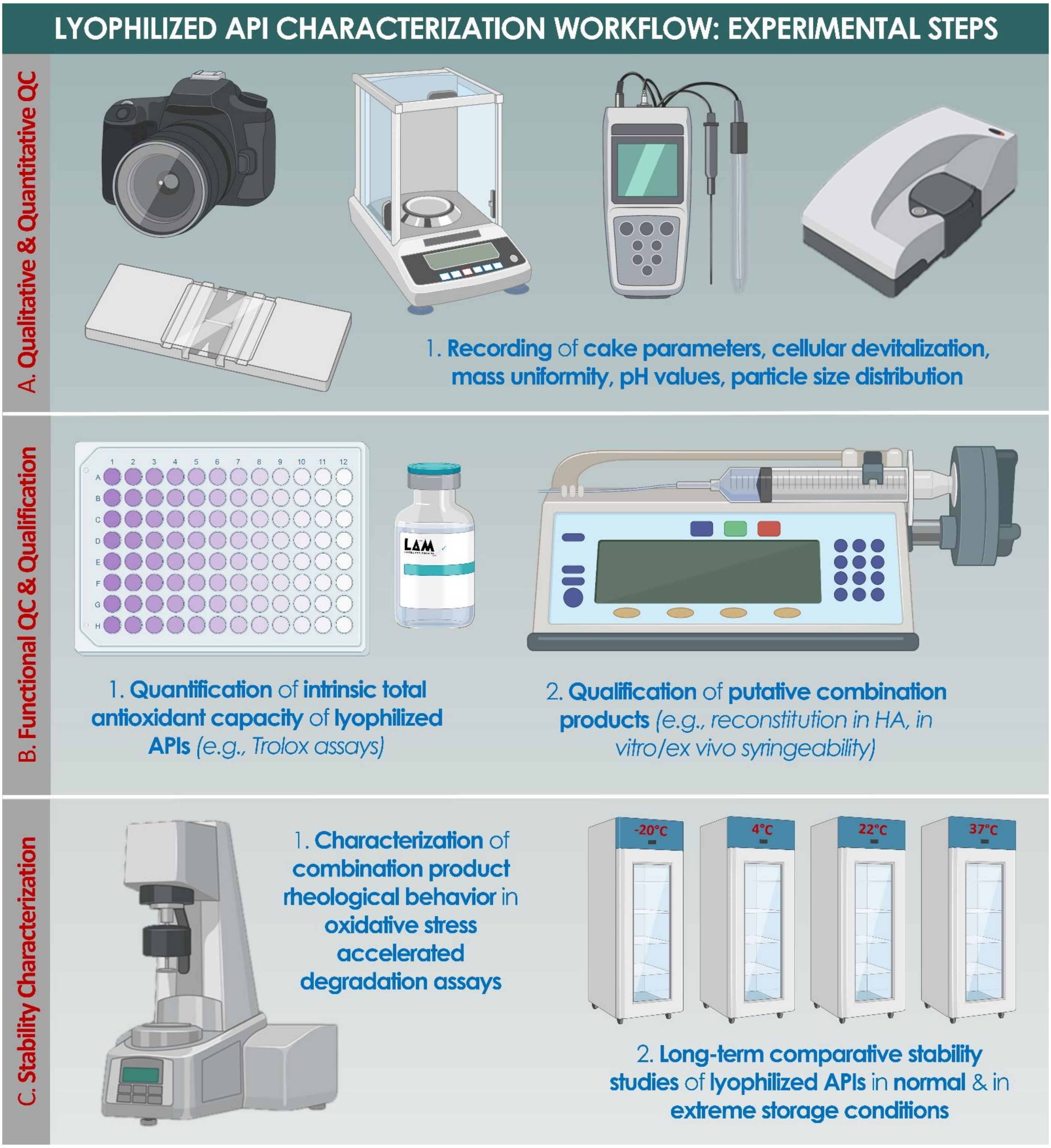

Figure S4. Experimental steps for lyophilized API characterization. (**A**) Qualitative and quantitative quality controls are performed in accordance with the requirements of the lyophilizate pharmaceutical form. (**B**) Specific functional parameters are investigated for the considered APIs (e.g., total antioxidant capacity), as well as for the combination products (i.e., APIs reconstituted in HA, e.g., syringeability in clinically relevant administration systems). The results of such studies yield important data relative to API and product quality attributes or mechanistic aspects, as well as technical qualification for product processing in a clinical setting (e.g., ease of administration by injection for the physician). (**C**) Stability assessments are performed in various settings for the exclusion of adverse impact yielding by the APIs on final product formulas following reconstitution, as well as during long-term storage. Experimental setups may comprise active and passive degradation or accelerated and normal conditions of storage (e.g., hydrogen peroxide challenge, high temperature storage). The results of such studies yield important data relative to API and product stability and are part of the shelf-life determination process. API, active pharmaceutical ingredient; HA, hyaluronic acid; QC, quality control.

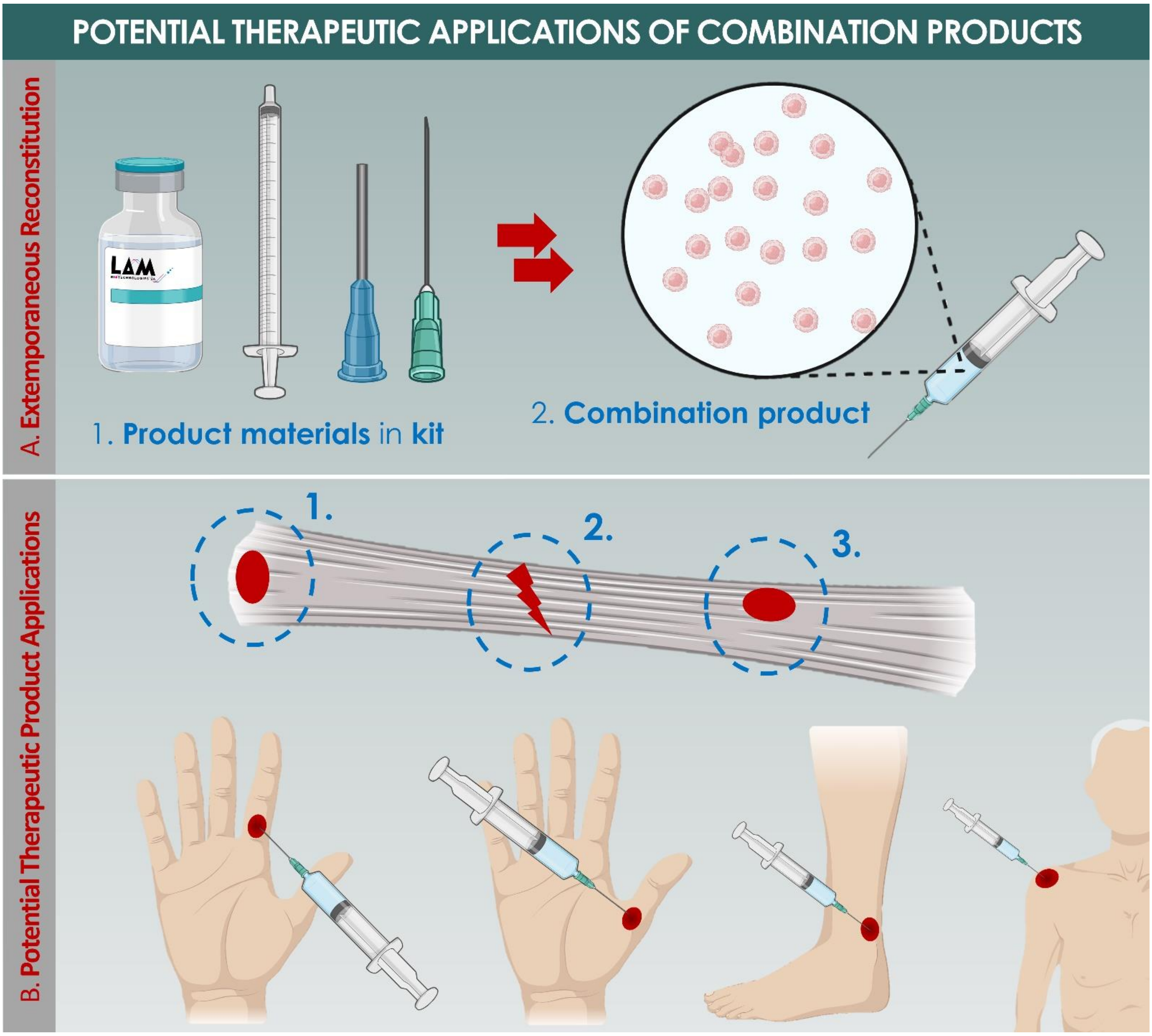

Figure S5. Potential therapeutic applications of extemporaneously reconstituted combination products. (**A**) Materials provided in a kit format may be used for appropriate reconstitution of the lyophilized API vial (e.g., whole cell lyophilizate) using HA (e.g., full combination product illustrated reconstitution workflow detailed in Figure S8). Following appropriate combination product preparation, the final administration system may be shortly stored or immediately transferred to the clinician. (**B**) Various potential therapeutic applications may be considered for the combination products at hand, such as tendon-ligament junction or tendinous tissue degenerative/inflammatory affections (e.g., evidenced anatomical sites 1 and 3) or partial tendon tissue ruptures (e.g., evidenced anatomical site 2). In terms of product indications, specific focus and further research shall be oriented toward tendinopathies principally affecting hand tendons, Achilles' tendons, and rotator cuff tendons. API, active pharmaceutical ingredient; HA, hyaluronic acid.

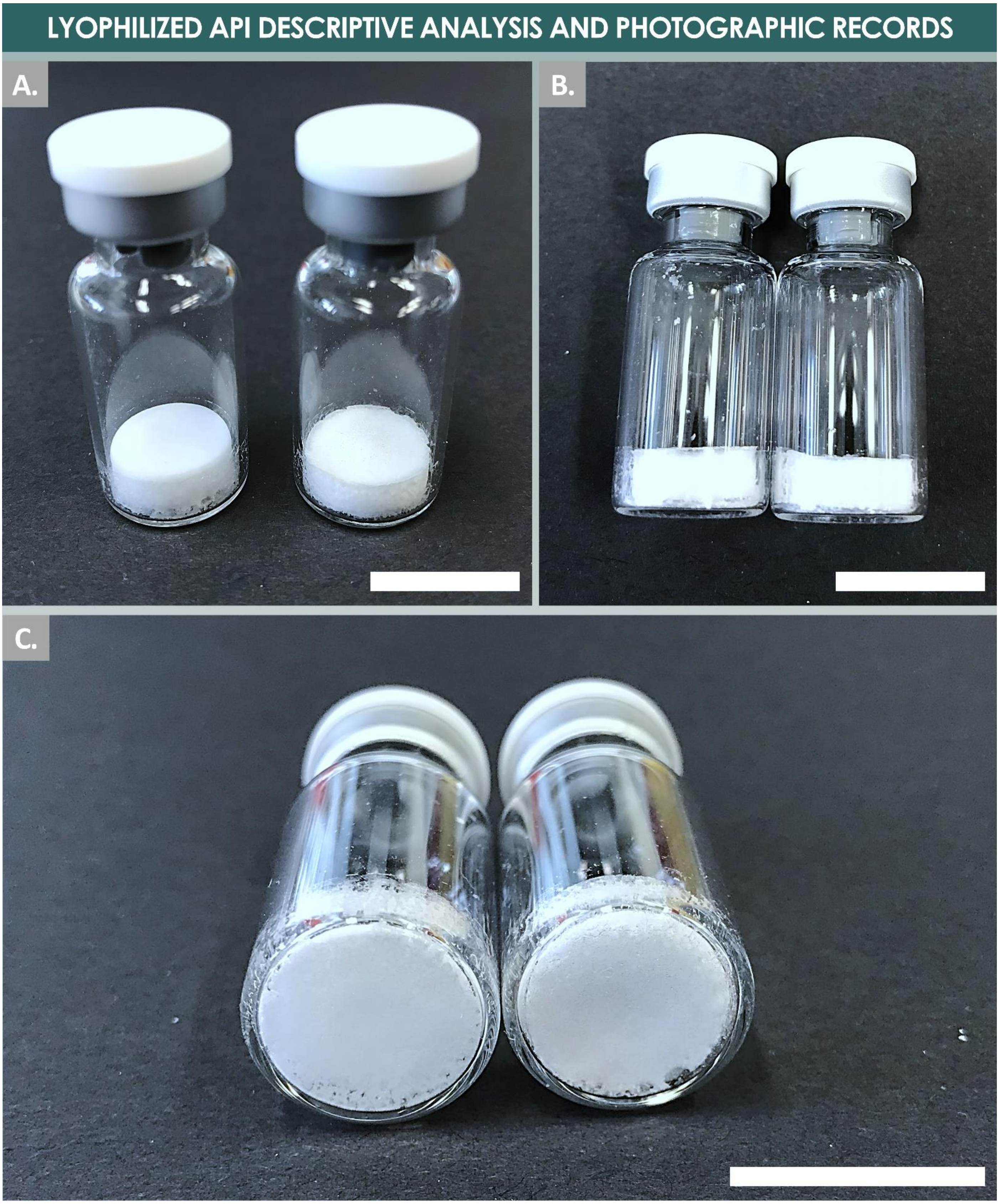

Figure S6. Lyophilized API (e.g., FE002-Ten progenitor cellular derivative materials) descriptive analysis and photographic records. Depicted API samples were lyophilized and stored at 4 °C for one month. (**A**) Aerial view of the whole cell (i.e., left) and cell lysate (i.e., right) lyophilizate API samples. (**B**) Side view of the whole cell (i.e., left) and cell lysate (i.e., right) lyophilizate API samples. (**C**) Bottom view of the whole cell (i.e., left) and cell lysate (i.e., right) lyophilizate API samples. Scale bars = 16 mm. API, active pharmaceutical ingredient.

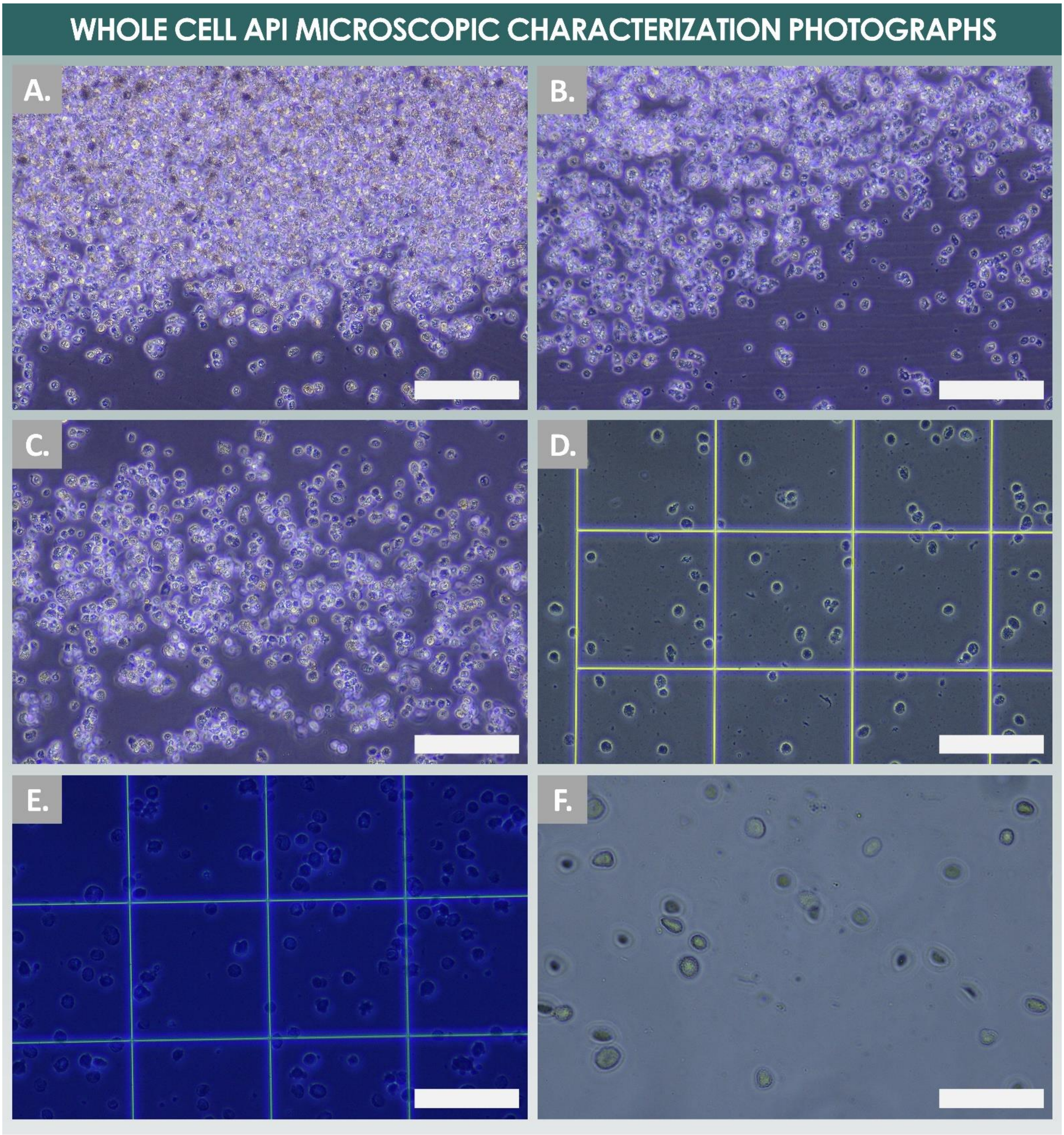

Figure S7. Whole cell lyophilized API microscopic characterization photographs of human progenitor tenocytes (e.g., FE002-Ten primary progenitor cells). (**A**) Contrast phase microscopic imaging of a reconstituted whole cell API lyophilizate in 150 µL of solvent. Scale bar = 200 µm. (**B**) Contrast phase microscopic imaging of a reconstituted and 5 × diluted whole cell API lyophilizate. Scale bar = 200 µm. (**C**) Contrast phase microscopic imaging of a reconstituted and 10 × diluted whole cell API lyophilizate. Scale bar = 200 µm. (**D**) Contrast phase microscopic imaging of a reconstituted whole cell API lyophilizate on a hemocytometer grid for manual particle enumeration. Scale bar = 200 µm. (**E**) Contrast phase microscopic imaging of a reconstituted whole cell API lyophilizate on a hemocytometer grid after sample staining with Trypan blue exclusion dye, for verification of cellular devitalization. Scale bar = 200 µm. (**F**) Contrast phase microscopic imaging of a whole cell API lyophilizate reconstituted in 2% HA (i.e., 1.2–1.5 MDa MW). Scale bar = 100 µm. API, active pharmaceutical ingredient; HA, hyaluronic acid; MDa, mega Daltons; MW, molecular weight.

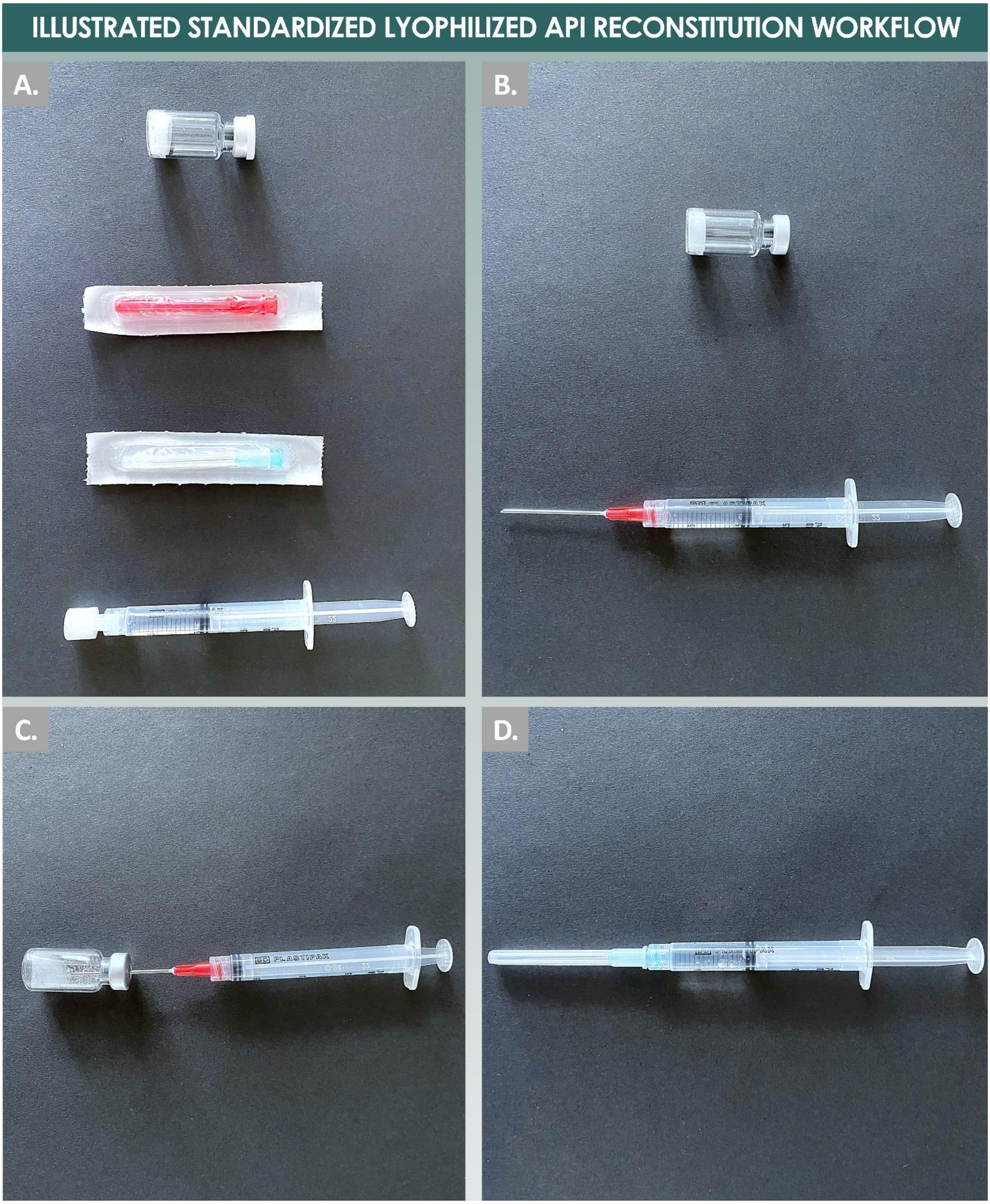

Figure S8. Lyophilized API standardized reconstitution workflow. (**A**) Typical kit contents may include the API vial, a loading and an administration needle, and the HA-based hydrogel in the capped administration syringe. All connections (i.e., syringe, needles, syringe cap) are mounted with Luer-Lok™ systems. (**B**) An 18G blunt fill needle is mounted on the administration syringe, for facilitated processing of the HA hydrogel. (**C**) The HA hydrogel is injected into the API vial, to allow appropriate reconstitution of the lyophilizate and suspension of the API materials. Once a homogenous preparation is obtained in the vial (i.e., waiting time > 5 min), the composite hydrogel formula is loaded in the administration syringe through the 18G blunt fill needle, for ease of product processing. (**D**) A small-bore product administration needle (e.g., 27G–34G) is finally mounted on the administration syringe in view of clinical application. API, active pharmaceutical ingredient; HA, hyaluronic acid.

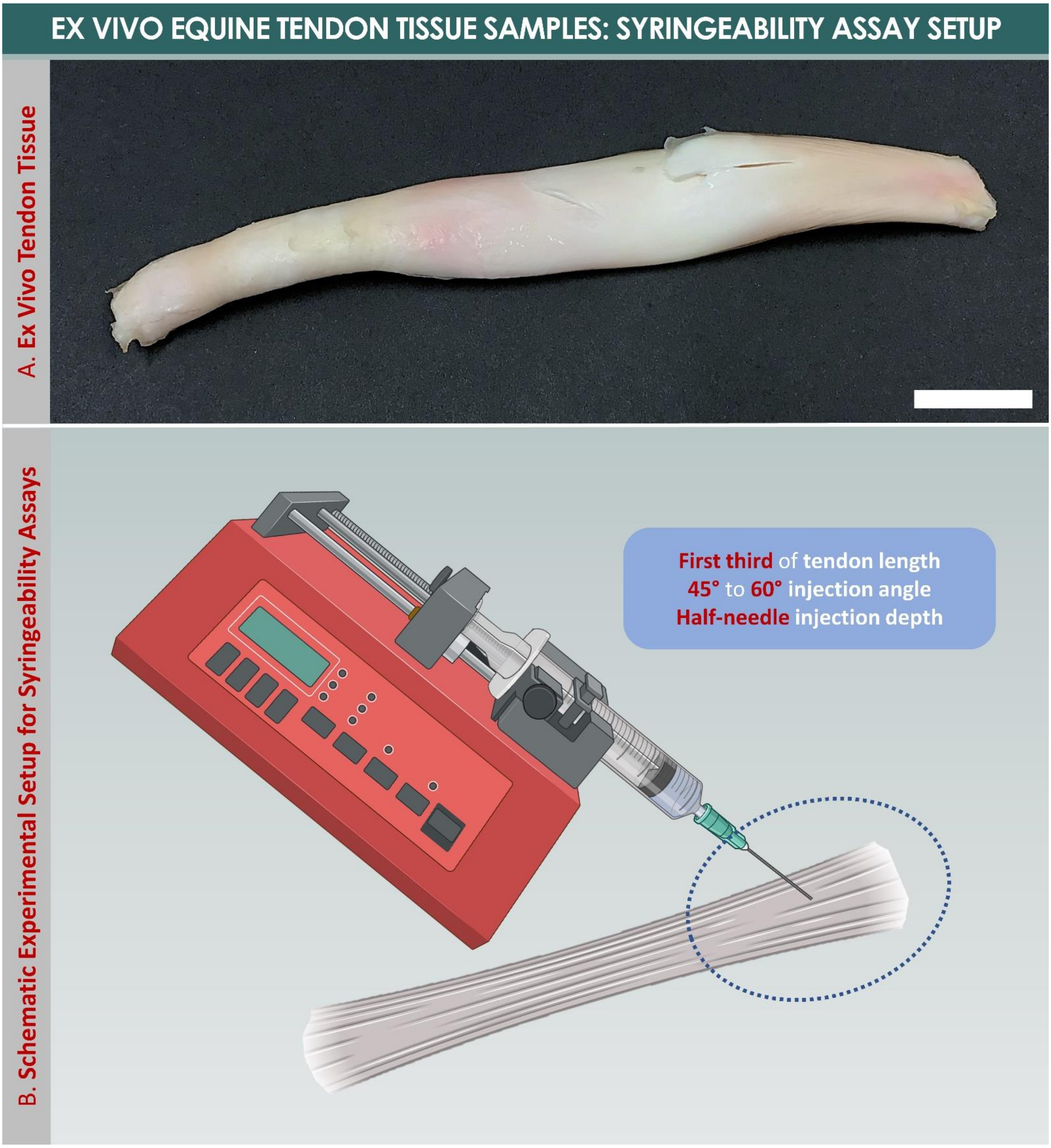

Figure S9. Experimental setup for the evaluation of syringeability of the combination product formulas in ex vivo settings. (**A**) Photographic illustration of an ex vivo equine tendon tissue sample used for the second part of the product syringeability assays. The total overall length of the tissue was of approximately 15 cm. Scale bar = 2 cm. (**B**) Schematic experimental setup of the second part of the product syringeability assays, with monitoring and recording of the force injection profiles. Technical specifications are provided for the assay setup and were consistently used for all the tendon tissue samples and product formulas (i.e., lyophilized APIs reconstituted in HA hydrogels). Injection needles presented a bore of 27G and a length of 13 mm. API, active pharmaceutical ingredient; HA, hyaluronic acid.

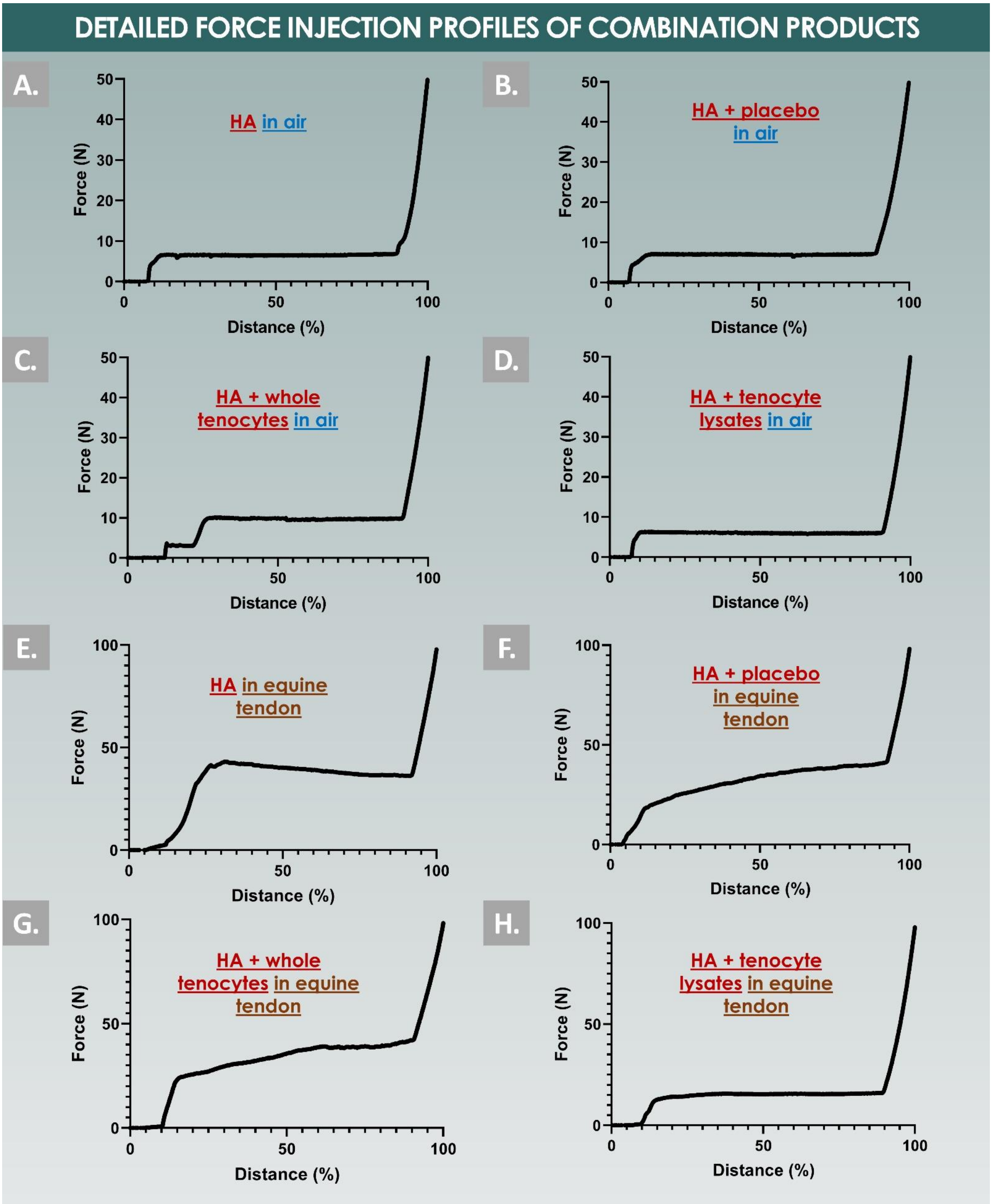

Figure S10. Detailed force injection profiles from the comparative syringeability assays performed in vitro and ex vivo for the combination products, as presented in Figure 6. (**A**) HA injected into air. (**B**) HA with the placebo API injected into air. (**C**) HA with the whole cell API injected into air. (**D**) HA with the cell lysate API injected into air. (**E**) HA injected into tendon. (**F**) HA with the placebo API injected into tendon. (**G**) HA with the whole cell API injected into tendon. (**H**) HA with the cell lysate API injected into tendon. API, active pharmaceutical ingredient; HA, hyaluronic acid; N, Newtons.

Supplementary Tables

Table S1. Quantitative results of the lyophilized progenitor cell derivative API particle size distribution analysis, following API reconstitution in HA hydrogels and following appropriate dilution using distilled water, but without addition of H_2O_2, as visually presented in Figure 5. The results indicated that the lyophilized cell lysates obtained by freeze-thaw were relatively smaller and less homogenous in terms of particle size distribution, as compared to the whole cell lyophilizate API group. The samples were diluted as appropriate for analysis. The measurements were performed with $n = 5$ and the results are presented as mean values with the corresponding standard deviations. API, active pharmaceutical ingredient; HA, hyaluronic acid.

**Parameters**	**Cell Lysate API**	**Whole Cell API**
Span values	3.816 ± 0.491	0.809 ± 0.044
Surface mean values D [3;2]	19.48 µm ± 1.19 µm	20.44 µm ± 0.59 µm
Volume mean diameters D [4;3]	38.62 µm ± 5.06 µm	22.32 µm ± 0.84 µm
Volume distributions (10%) Dv (10)	9.89 µm ± 0.36 µm	14.44 µm ± 0.25 µm
Volume distribution medians (50%) Dv (50)	21.56 µm ± 2.01 µm	21.34 µm ± 0.71 µm
Volume distributions (90%) Dv (90)	91.72 µm ± 9.52 µm	31.72 µm ± 1.70 µm

Table S2. Quantitative results of Tukey's multiple comparison test, in relation with the endpoint (i.e., experimental values recorded at the 10 min timepoint) rheological profiles presented in Figure 1. The HA group represented the HA control. The data were obtained after a repeated measures two-way ANOVA analysis was performed. Non-significative differences corresponded to a *p*-value > 0.05. A significance level described by one asterisk "*" corresponded to a *p*-value between 0.01 and 0.05, and a significance level described by two asterisks "**" corresponded to a *p*-value between 0.001 and 0.01. HA, hyaluronic acid; ns, non-significant; Pa, Pascals; Pa·s, Pascal seconds.

**Rheological Parameters**	**Compared Groups**	**Mean Absolute Difference** (Pa or Pa·s)	**Adjusted *p*-Value**	**Significance Level**
**Storage moduli (G′)**	HA / Placebo	-0.9197	0.0022	**
	HA / Whole cell	-2.0130	0.0082	**
	HA / Cell lysate	-1.7870	0.0536	ns
	Placebo / Whole cell	-1.0930	0.0344	*
	Placebo / Cell lysate	-0.8678	0.2138	ns
	Whole cell / Cell lysate	0.2256	0.8987	ns
**Loss moduli (G″)**	HA / Placebo	-1.9710	0.0093	**
	HA / Whole cell	-2.8040	0.0031	**
	HA / Cell lysate	-2.6550	0.0608	ns
	Placebo / Whole cell	-0.8336	0.1516	ns
	Placebo / Cell lysate	-0.6839	0.5989	ns
	Whole cell / Cell lysate	0.1498	0.9909	ns
**Complex viscosity (η*)**	HA / Placebo	-0.3367	0.0077	**
	HA / Whole cell	-0.5211	0.0038	**
	HA / Cell lysate	-0.4850	0.0637	ns
	Placebo / Whole cell	-0.1844	0.1119	ns
	Placebo / Cell lysate	-0.1483	0.5045	ns
	Whole cell / Cell lysate	0.0361	0.9803	ns

Table S3. Quantitative results of Tukey's multiple comparison test, in relation with the endpoint (i.e., experimental values recorded at the 180 min timepoint) rheological profiles presented in Figure 2. The HA group represented the HA control. The data were obtained after a repeated measures two-way ANOVA analysis was performed. A significance level described by one asterisk "*" corresponded to a *p*-value between 0.01 and 0.05, a significance level described by two asterisks "**" corresponded to a *p*-value between 0.001 and 0.01, a significance level described by three asterisks "***" corresponded to a *p*-value between 0.0001 and 0.001, and a significance level described by four asterisks "****" corresponded to a *p*-value < 0.0001. HA, hyaluronic acid; Pa, Pascals; Pa·s, Pascal seconds.

**Rheological Parameters**	**Compared Groups**	**Mean Absolute Difference** (Pa or Pa·s)	**Adjusted *p*-Value**	**Significance Level**
**Storage moduli (G′)**	HA / Placebo	-0.8333	0.0002	***
	HA / Whole cell	-2.5570	0.0025	**
	HA / Cell lysate	-1.7470	< 0.0001	****
	Placebo / Whole cell	-1.7230	0.0041	**
	Placebo / Cell lysate	-0.9133	< 0.0001	****
	Whole cell / Cell lysate	0.8100	0.0141	*
**Loss moduli (G″)**	HA / Placebo	-2.4670	< 0.0001	****
	HA / Whole cell	-4.6030	< 0.0001	****
	HA / Cell lysate	-3.3870	< 0.0001	****
	Placebo / Whole cell	-2.1370	0.0001	***
	Placebo / Cell lysate	-0.9200	< 0.0001	****
	Whole cell / Cell lysate	1.2170	0.0030	**
**Complex viscosity (η*)**	HA / Placebo	-0.4100	< 0.0001	****
	HA / Whole cell	-0.8233	0.0002	***
	HA / Cell lysate	-0.5933	< 0.0001	****
	Placebo / Whole cell	-0.4133	0.0022	**
	Placebo / Cell lysate	-0.1833	< 0.0001	****
	Whole cell / Cell lysate	0.2300	0.0065	**

Table S4. Proteomic database entries relative to the quantified API proteins [1] listed in Table 1. Data were gathered from the UniProt online database (www.uniprot.org, accessed 28.10.2021). Gene ontology entries (i.e., for molecular function and biological processes, respectively) with potential implications for tendons and related physiological or pathological processes were collected and are listed hereunder. API, active pharmaceutical ingredient; GO, gene ontology; NA, non-applicable.

**Protein Abbreviation** Protein Name	**Entry Number**	**GO Molecular Function Entries**	**GO Biological Process Entries**
**MMP-2** 72 kDa type IV collagenase	P08253	Endopeptidase activity; metalloendopeptidase activity; metallopeptidase activity; serine-type endopeptidase activity; zinc ion binding.	Angiogenesis; blood vessel maturation; cellular protein metabolic process; cellular response to amino acid stimulus; cellular response to reactive oxygen species; collagen catabolic process; cytokine-mediated signaling pathway; endodermal cell differentiation; extracellular matrix disassembly; extracellular matrix organization; proteolysis; response to hypoxia; tissue remodeling.
**TIMP-2** Metalloproteinase inhibitor 2	P16035	Enzyme inhibitor activity; integrin binding; metalloendopeptidase inhibitor activity; peptidase inhibitor activity; protease binding; zinc ion binding.	Aging; extracellular matrix disassembly; negative regulation of cell population proliferation; negative regulation of endopeptidase activity; negative regulation of membrane protein ectodomain proteolysis; negative regulation of metallopeptidase activity; negative regulation of mitotic cell cycle; negative regulation of Ras protein signal transduction; positive regulation of MAPK cascade; response to cytokine; response to drug; response to hormone; response to organic substance.
**sEGFR** Soluble epidermal growth factor receptor	NA	NA	NA
**TIMP-1** Metalloproteinase inhibitor 1	P01033	Cytokine activity; enzyme inhibitor activity; growth factor activity; metalloendopeptidase inhibitor activity; peptidase inhibitor activity; protease binding; zinc ion binding.	Aging; cell activation; cellular protein metabolic process; connective tissue replacement involved in inflammatory response wound healing; cytokine-mediated signaling pathway; extracellular matrix disassembly; negative regulation of apoptotic process; negative regulation of catalytic activity; negative regulation of endopeptidase activity; negative regulation of membrane protein ectodomain proteolysis; negative regulation of metallopeptidase activity; platelet degranulation; positive regulation of cell population proliferation; post-translational protein modification; regulation of integrin-mediated signaling pathway; response to cytokine; response to hormone; response to organic substance; response to peptide hormone.
**sgp130** Soluble gp130	NA	NA	NA

**FGF-2** Fibroblast growth factor 2	P09038	Chemoattractant activity; chemokine binding; cytokine activity; fibroblast growth factor receptor binding; growth factor activity; heparin binding; identical protein binding; integrin binding; nuclear receptor coactivator activity; receptor-receptor interaction.	Cell differentiation; cell migration involved in sprouting angiogenesis; chemotaxis; cytokine-mediated signaling pathway; extracellular matrix organization; fibroblast growth factor receptor signaling pathway; hyaluronan catabolic process; negative regulation of cell death; negative regulation of fibroblast growth factor receptor signaling pathway; negative regulation of fibroblast migration; negative regulation of gene expression; negative regulation of wound healing; paracrine signaling; positive regulation of angiogenesis; positive regulation of cell division; positive regulation of cell fate specification; positive regulation of cell population proliferation; positive regulation of DNA biosynthetic process; positive regulation of endothelial cell chemotaxis; positive regulation of endothelial cell chemotaxis to fibroblast growth factor; positive regulation of endothelial cell migration; positive regulation of endothelial cell proliferation; positive regulation of gene expression; positive regulation of protein phosphorylation; regulation of angiogenesis; regulation of cell migration; release of sequestered calcium ion into cytosol; signal transduction; somatic stem cell population maintenance; stem cell proliferation; wound healing.
**HGF** Hepatocyte growth factor	P14210	Chemoattractant activity; growth factor activity; identical protein binding; protein-containing complex binding; serine-type endopeptidase activity.	Cell chemotaxis; cell morphogenesis; cellular response to hepatocyte growth factor stimulus; cytokine-mediated signaling pathway; hyaluronan metabolic process; mitotic cell cycle; negative regulation of apoptotic process; negative regulation of autophagy; negative regulation of hydrogen peroxide-mediated programmed cell death; negative regulation of inflammatory response; negative regulation of interleukin-6 production; negative regulation of release of cytochrome C from mitochondria; platelet degranulation; positive regulation of angiogenesis; positive regulation of cell migration; positive regulation of DNA biosynthetic process; positive regulation of interleukin-10 production; positive regulation of protein phosphorylation; positive regulation of transcription by RNA polymerase II.
**sTNFRI** Soluble tumour necrosis factor receptor type I	NA	NA	NA
**MMP-13** Collagenase 3	P45452	Calcium ion binding; collagen binding; endopeptidase activity; metalloendopeptidase activity; zinc ion binding.	Collagen catabolic process; extracellular matrix disassembly; extracellular matrix organization; proteolysis.
**IL-1Ra** Interleukin-1 receptor antagonist protein	P18510	Cytokine activity; interleukin-1 receptor binding; interleukin-1 receptor antagonist activity.	Acute-phase response; cytokine-mediated signaling pathway; immune response; inflammatory response; inflammatory response to antigenic stimulus; insulin secretion; interleukin-1-mediated signaling pathway; lipid metabolic process; negative regulation of heterotypic cell-cell adhesion; negative regulation of interleukin-1-mediated signaling pathway; response to glucocorticoid.

**FST**<br>Follistatin	P19883	Activin binding; activin receptor antagonist activity; heparan sulfate proteoglycan binding.	BMP signaling pathway; cell differentiation; negative regulation of activin receptor signaling pathway; negative regulation of cell differentiation; negative regulation of transcription by RNA polymerase II.
**MMP-7**<br>Matrilysin	P09237	Endopeptidase activity; heparin binding; metalloendopeptidase activity; metallopeptidase activity; zinc ion binding.	Cellular response to mechanical stimulus; collagen catabolic process; extracellular matrix disassembly; extracellular matrix organization; membrane protein ectodomain proteolysis; membrane protein intracellular domain proteolysis; positive regulation of cell migration; proteolysis; response to nutrient levels.
**FGF-1**<br>Fibroblast growth factor 1	P05230	Fibroblast growth factor receptor binding; growth factor activity; heparin binding; Hsp70 protein binding; integrin binding; S100 protein binding.	Activation of MAPK activity; activation of protein kinase B activity; angiogenesis; cell differentiation; cellular response to heat; fibroblast growth factor receptor signaling pathway; positive regulation of angiogenesis; positive regulation of cell division; positive regulation of cell migration; positive regulation of cell population proliferation; positive regulation of gene expression; positive regulation of intracellular signal transduction; positive regulation of protein phosphorylation; positive regulation of sprouting angiogenesis; positive regulation of transcription by RNA polymerase II; regulation of cell migration; regulation of endothelial cell chemotaxis to fibroblast growth factor; signal transduction; wound healing.
**IL-23**<br>Interleukin-23	NA	NA	NA
**ENG**<br>Endoglin	P17813	Activin binding; coreceptor activity; galactose binding; glycosaminoglycan binding; identical protein binding; protein homodimerization activity; transforming growt factor beta-activated receptor activity; transforming growth factor beta binding; transmembrane signaling receptor activity; transforming growth factor beta receptor binding.	Angiogenesis; cell adhesion; cell chemotaxis; cell migration; cell motility; cellular response to mechanical stimulus; detection of hypoxia; extracellular matrix constituent secretion; extracellular matrix disassembly; negative regulation of cell migration; negative regulation of endothelial cell proliferation; negative regulation of gene expression; negative regulation of nitric-oxide synthase activity; negative regulation of protein autophosphorylation; negative regulation of transcription by RNA polymerase II; negative regulation of transforming growth factor beta receptor signaling pathway; positive regulation of angiogenesis; positive regulation of collagen biosynthetic process; positive regulation of gene expression; regulation of cell adhesion; regulation of cell population proliferation; regulation of phosphorylation; response to corticosteroid; response to drug; response to hypoxia; vasculogenesis; venous blood vessel morphogenesis; wound healing.
**MDC/CCL22**<br>C-C motif chemokine 22	O00626	CCR chemokine receptor binding; chemokine activity.	Cell-cell signaling; cellular response to interferon-gamma; cellular response to interleukin-1; cellular response to tumor necrosis factor; chemokine-mediated signaling pathway; chemotaxis; cytokine-mediated signaling pathway; G protein-coupled receptor signaling pathway; immune response; inflammatory response; killing of cells of other organism; lymphocyte chemotaxis; monocyte

			chemotaxis; neutrophil chemotaxis; positive regulation of ERK1 and ERK2 cascade; positive regulation of GTPase activity; response to virus; signal transduction.
**Flt-3L** Fms-related tyrosine kinase 3 ligand	P49771	Cytokine activity; receptor tyrosine kinase binding; signaling receptor binding.	Cytokine-mediated signaling pathway; positive regulation of cell population proliferation; positive regulation of natural killer cell proliferation; positive regulation of protein kinase B signaling; signal transduction.
**VEGF-A** Vascular endothelial growth factor A	P15692	Chemoattractant activity; cytokine activity; extracellular matrix binding; fibronectin binding; growth factor activity; heparin binding; identical protein binding; neuropilin binding; platelet-derived growth factor receptor binding; protein homodimerization activity; receptor ligand activity; vascular endothelial growth factor receptor binding.	Activation of protein kinase activity; angiogenesis; basophil chemotaxis; cell maturation; cell migration involved in sprouting angiogenesis; cellular response to hypoxia; cellular response to vascular endothelial growth factor stimulus; cellular stress response to acid chemical; cytokine-mediated signaling pathway; endothelial cell chemotaxis; macrophage differentiation; monocyte differentiation; negative regulation of apoptotic process; negative regulation of gene expression; negative regulation of transcription by RNA polymerase II; platelet degranulation; positive chemotaxis; positive regulation of angiogenesis; positive regulation of cell adhesion; positive regulation of cell division; positive regulation of cell migration; positive regulation of cell population proliferation; positive regulation of cellular component movement; positive regulation of cold-induced thermogenesis; positive regulation of endothelial cell chemotaxis by VEGF-activated vascular endothelial growth factor receptor signaling pathway; positive regulation of endothelial cell migration; positive regulation of endothelial cell proliferation; positive regulation of epithelial cell proliferation; positive regulation of gene expression; positive regulation of leukocyte migration; positive regulation of mast cell chemotaxis; positive regulation of mesenchymal cell proliferation; positive regulation of protein phosphorylation; positive regulation of vascular permeability; regulation of cell shape; regulation of nitric oxide mediated signal transduction; response to hypoxia; surfactant homeostasis; vascular wound healing; vasculogenesis.
**MCP-1/CCL2** C-C motif chemokine 2	P13500	CCR2 chemokine receptor binding; CCR chemokine receptor binding; chemokine activity; protein kinase activity; signaling receptor binding.	Angiogenesis; cell adhesion; cell surface receptor signaling pathway; cellular homeostasis; cellular response to fibroblast growth factor stimulus; cellular response to interferon-gamma; cellular response to interleukin-1; cellular response to lipopolysaccharide; cellular response to organic cyclic compound; cellular response to tumor necrosis factor; chemokine-mediated signaling pathway; chemotaxis; cytokine-mediated signaling pathway; cytoskeleton organization; G protein-coupled receptor signaling pathway; helper T cell extravasation; humoral immune response; inflammatory response; lipopolysaccharide-mediated signaling pathway; lymphocyte chemotaxis; macrophage chemotaxis; monocyte chemotaxis; negative regulation of G1/S transition of mitotic cell cycle; negative regulation of natural killer cell chemotaxis; positive regulation of apoptotic cell clearance; positive regulation of calcium ion import; positive regulation of endothelial cell apoptotic process; positive regulation of ERK1 and ERK2 cascade; positive regulation of GTPase activity; positive regulation of nitric-oxide synthase biosynthetic process; positive regulation of NMDA glutamate receptor activity; positive regulation of T cell activation; protein kinase B signaling; protein phosphorylation; receptor signaling

			pathway via JAK-STAT; regulation of cell shape; response to bacterium; sensory perception of pain; signal transduction.
**sIL-6R** Soluble IL-6 receptor	NA	NA	NA

[1] The entries in the table represent the 20 most abundant proteins detected in the bulk API samples. Other proteins detected in the bulk API samples in quantities between 0 pg/mg and 50 pg/mg (i.e., expressed in relative protein quantity in the total FE002-Ten progenitor cell lysate, listed in decreasing relative quantitative order of appearance) are listed in Table S5.

Table S5. Proteomic characterization results of the bulk cellular materials (i.e., FE002-Ten primary progenitor tenocyte bulk lysate) used for lyophilized API manufacture. The 20 most abundant proteins were reported in Table 1 and in Table S4. Other proteins, detected in quantities between 0.00 pg/mg and 50.00 pg/mg, are presented hereunder in decreasing order of appearance as classified by relative detected quantities. Calculated protein quantities in the API unitary doses were listed for each entry. The relative protein quantity detected and normalized to the unfractionated cell lysate total protein content was expressed in pg of the specified protein per mg of the whole cell lysate total protein content. The calculated quantity of proteins in an API unitary dose (i.e., corresponding to 1.5 × 10^6 cell unit equivalents) was expressed in pg of the specified protein per lyophilizate vial. API, active pharmaceutical ingredient.

Protein Abbreviated Name (Protein Full Name)	Normalized Relative Protein Quantity in Cell Lysate (pg/mg)	Calculated Protein Quantity in an API Unitary Dose (pg/vial)
IFN-β (Interferon beta)	36.84	10.45
MMP-8 (Matrix metalloproteinase-8)	35.90	10.18
IL-16 (Interleukin 16)	34.21	9.70
VEGF-C (Vascular endothelial growth factor C)	29.67	8.42
sVEGFR3 (Soluble vascular endothelial growth factor receptor 3)	28.54	8.10
CX3CL1 (Fractalkine)	27.33	7.75
MMP-9 (Matrix metalloproteinase-9)	26.08	7.40
sVEGFR1 (Soluble vascular endothelial growth factor receptor 1)	22.52	6.39
MMP-1 (Matrix metalloproteinase-1)	22.25	6.31
sVEGFR2 (Soluble vascular endothelial growth factor receptor 2)	21.97	6.23
sTNFRII (Soluble tumor necrosis factor receptor type II)	21.09	5.98
TGF-β2 (Transforming growth factor beta-2)	19.99	5.67
MMP-3 (Matrix metalloproteinase-3)	19.79	5.61
GRO-α (Chemokine growth-regulated protein alpha)	15.55	4.41
IL-29 (Interleukin 29)	15.28	4.34
MMP-10 (Matrix metalloproteinase-10)	14.71	4.17
MMP-12 (Matrix metalloproteinase-12)	13.42	3.81
PDGF-BB (Platelet-derived growth factor-BB)	11.85	3.36
IL-18 (Interleukin 18)	11.75	3.33
sIL-4R (Soluble interleukin-4 receptor)	8.63	2.45
6Ckine (C-C motif chemokine ligand 21)	7.20	2.04
MCP-3 (Monocyte chemotactic protein-3)	5.82	1.65
IL-6 (Interleukin 6)	4.79	1.36
IFN-α (Interferon alpha)	4.63	1.31
MCP-2 (Monocyte chemotactic protein-2)	4.58	1.30
IP-10 (Serum IFN-γ-induced protein 10)	4.24	1.20
VEGF-D (Vascular endothelial growth factor-D)	4.13	1.17
MCP-4 (Human monocyte chemoattractant protein-4)	3.89	1.10
sIL-2RA (Soluble interleukin 2 receptor alpha)	3.62	1.03
ET-1 (Endothelin-1)	3.22	0.91
IL-4 (Interleukin 4)	2.70	0.77

MIP-1α (Macrophage inflammatory protein-1 alpha)	2.63	0.75
G-CSF (Granulocyte colony-stimulating factor)	2.10	0.60
sIL-1RI (Soluble interleukin-1 receptor type I)	2.10	0.60
TGF-β1 (Transforming growth factor beta 1)	2.06	0.59
sCD30 (Soluble CD30)	2.05	0.58
SDF-1A+B (Stromal cell-derived factor-1 alpha and beta)	1.95	0.55
IL-20 (Interleukin 20)	1.83	0.52
IL-21 (Interleukin 21)	1.81	0.51
IL-8 (Interleukin 8)	1.62	0.46
Ang2 (Angiopoietin-2)	1.59	0.45
IL-12 p40 (Interleukin-12 subunit beta)	1.45	0.41
MIP-1β (Macrophage inflammatory protein-1 beta)	1.35	0.38
GM-CSF (Granulocyte-macrophage colony-stimulating factor)	1.09	0.31
IFN-γ (Interferon gamma)	1.08	0.31
IL-13 (Interleukin 13)	0.91	0.26
IL-7 (Interleukin 7)	0.72	0.21
TGF-β3 (Transforming growth factor beta-3)	0.66	0.19
PDGF-AA (Platelet-derived growth factor AA)	0.61	0.17
IL-15 (Interleukin 15)	0.61	0.17
EGF (Epidermal growth factor)	0.58	0.16
sRAGE (Soluble receptor for advanced glycation end products)	0.57	0.16
IFN-ω (Interferon omega)	0.50	0.14
PLGF (Placental growth factor)	0.49	0.14
TSLP (Thymic stromal lymphopoietin)	0.46	0.13
HB-EGF (Heparin binding EGF like growth factor)	0.45	0.13
RANTES (Regulated upon activation, normal T cell expressed and presumably secreted)	0.45	0.13
IL-5 (Interleukin 5)	0.44	0.13
BMP-9 (Bone morphogenetic protein 9)	0.43	0.12
TNF-β (Tumor necrosis factor-beta)	0.43	0.13
TRAIL (TNF-related apoptosis inducing ligand)	0.41	0.12
CCL11 (Eotaxin-1)	0.25	0.07
TARC (Thymus- and activation-regulated chemokine)	0.22	0.06
IL-2 (Interleukin 2)	0.19	0.05
IL-9 (Interleukin 9)	0.19	0.05
CTACK (Cutaneous T cell-attracting chemokine)	0.19	0.05
CCL24 (Eotaxin-2)	0.17	0.05
IL-1β (Interleukin-1beta)	0.17	0.05
IFN-α (Interferon alpha)	0.17	0.05
IL-33 (Interleukin 33)	0.16	0.05
IL-1α (Interleukin-1alpha)	0.16	0.05
CCL1 (I-309)	0.16	0.05

CXCL13 (BCA-1)	0.15	0.04
SCF (Skp, Cullin, F-box containing complex)	0.13	0.04
IL-17A (Interleukin 17A)	0.11	0.03
IL-10 (Interleukin 10)	0.08	0.02
TGF-α (Transforming growth factor alpha)	0.03	< 0.01
IL-12 p70 (Interleukin 12 p70)	0.01	< 0.01
IL-3 (Interleukin 3)	0.01	< 0.01
